# Transferability, development of simple sequence repeat (SSR) markers and application to the analysis of genetic diversity and population structure of the African fan palm (*Borassus aethiopum* Mart.) in Benin

**DOI:** 10.1186/s12863-020-00955-y

**Published:** 2020-12-03

**Authors:** Mariano Joly Kpatènon, Kolawolé Valère Salako, Sylvain Santoni, Leila Zekraoui, Muriel Latreille, Christine Tollon-Cordet, Cédric Mariac, Estelle Jaligot, Thierry Beulé, Kifouli Adéoti

**Affiliations:** 1 Laboratoire de Microbiologie et de Technologie Alimentaire (LAMITA), Faculté des Sciences et Techniques, Université d’Abomey-Calavi, Cotonou, Bénin; 2 Biodiversité et Ecologie des Plantes (BDEP), Faculté des Sciences et Techniques, Université d’Abomey-Calavi, Cotonou, Bénin; 3 DIADE, Univ Montpellier, IRD, Montpellier, France; 4 Laboratoire de Biomathématiques et d’Estimations Forestières (LABEF), Faculté des Sciences Agronomiques, Université d’Abomey-Calavi, Cotonou, Bénin; 5 AGAP, Univ Montpellier, CIRAD, INRAE, Montpellier SupAgro, Montpellier, France; 6 CIRAD, UMR DIADE, Montpellier, France

**Keywords:** *Borassus aethiopum*, Genetic diversity, Microsatellite, Marker transferability, High-throughput sequencing, Simple sequence repeat, Under-studied species

## Abstract

**Background:**

In Sub-Saharan Africa, *Borassus aethiopum* Mart. (African fan palm) is an important non-timber forest product-providing palm that faces multiple anthropogenic threats to its genetic diversity. However, this species is so far under-studied, which prevents its sustainable development as a resource. The present work is a first attempt at characterizing the genetic diversity and population structure of *B. aethiopum* across nine collection sites spanning the three climatic regions of Benin, West Africa, through the use of microsatellite markers.

**Results:**

During a first phase we relied on the reported transferability of primers developed in other palm species. We find that, in disagreement with previously published results, only 22.5% of the markers tested enable amplification of *B. aethiopum* DNA and polymorphism detection is very low.

In a second phase, we generated a *B. aethiopum*-specific genomic dataset through high-throughput sequencing and used it for the de novo detection of microsatellite loci. Among the primer pairs targeting these, 11 detected polymorphisms and were further used for analyzing genetic diversity. Across the nine sites, expected heterozygosity (He) ranges from 0.263 to 0.451 with an overall average of 0.354, showing a low genetic diversity. Analysis of molecular variance (AMOVA) shows that within-site variation accounts for 53% of the genetic variation. Accordingly, the low number of migrants and positive values of the fixation index (F) in sites from both the Central (Sudano-Guinean) and the Southern (Guinean) climatic regions suggest limited gene flow between sites. The global correlation between genetic and geographic distances is weak; however, our clustering analyses indicate that *B. aethiopum* palms from Savè (Center) are genetically more similar to those from the North than to samples from other Central sites.

**Conclusions:**

In the light of our results, we discuss the use of inter-species transfer vs. de novo development of microsatellite markers in genetic diversity analyses targeting under-studied species, and suggest future applications for our molecular resources. We propose that, while prominent short-range pollen and seed dispersal in Benin explain most of our results, gene flux between the Central and Northern regions, as a result of animal and/or human migrations, might underlie the Savè discrepancy.

**Supplementary Information:**

The online version contains supplementary material available at 10.1186/s12863-020-00955-y.

## Background

Many plant species remain under-studied due to their low economic importance, complicated biology and/or the absence of available genome sequence information. Upon initiating a research project aimed at characterizing the genetic diversity of such a species, researchers may be confronted with the situation that some resources can be found in related taxa. In such cases, the first step is often to assess whether some of these resources, such as molecular markers, can be used to study the new species. Provided that the “source” species display enough genetic similarities to the “target” species and that marker transferability has been previously assessed, this first step may lead to quick progress in a cost-effective manner. Often, transferring markers between species is seen as a smarter investment than developing and testing new markers, especially if limited funding is available [1, 2].

Over the last three decades, molecular markers have been widely used to study genetic variation among and within populations of various plant species [3–7]. Among the different types of markers that are available, microsatellites or simple sequence repeats (SSRs) are often selected due to their high mutation frequency, which ranges from 10^− 2^ to 10^− 6^ nucleotides per locus per generation [2, 8] and generates multiple allelic forms, and their co-dominant nature. The combination of both characteristics makes them sensitive tools for the assessment of genetic diversity among species, determination of population structure, phylogenetic reconstruction, genetic mapping, evolutionary analyses, and molecular breeding [9–12]. From a practical perspective, the popularity of SSRs is also related to their low resource requirements (i.e. technical skills, laboratory equipments and consumables) that enable their easy implementation and the reproducibility of results in most research environments [2, 8]. However, the steps leading to the development of functional SSR markers, namely the initial identification of microsatellite loci, primer selection and assessment of amplification/polymorphism detection, require some prior knowledge of the genome of the target species and may prove to be expensive and time-consuming [11, 13]. In order to overcome this difficulty, approaches relying on the transfer of SSR markers between species or genera have therefore been implemented. They have been successful in many instances, as documented across *Prunus* species and among members of the Rosaceae family [14, 15]; between species of the *Hevea* genus and to other Euphorbiaceae [16]; among Lamiaceae [17]; among Legumes belonging to the *Vicia* genus [18] and from the *Phaseolus* genus to *Vigna* [19]. In other cases, the ever-increasing affordability of high-throughput sequencing technologies and the development of dedicated bioinformatics data mining tools have enabled the identification of microsatellite loci and the development of SSR markers, including in non-model plant species with limited or no background genetic information [20–23].

*Borassus aethiopum* Mart., also known as ron palm, toddy palm or African fan palm, is a dioecious species belonging to the Arecaceae family. It is widely distributed across West and Central Africa, where it is present as wild populations [24]. The species is classified as a non-timber forest products (NTFPs)-providing plant, since different parts of the plant are used for various purposes by local populations [24, 25]. In Benin (West Africa) for instance, 121 different uses distributed in seven categories (medicinal, handicrafts, food, construction, firewood, ceremonies and rituals) have been reported for the species [26]. Among these, the consumption of ripe fruits (fresh or roasted) and hypocotyls as food, the use of the weather- and pest-resistant stipe as construction wood and that of leaves and petioles in handicrafts, are the most widespread in local populations [26–28]. These different products are also sold in markets, mostly by women, to whom they provide additional income: it is indeed estimated that in Benin, sales of hypocotyls alone may represent 50% to nearly three times the minimum wage of 40,000 CFA Francs (ca. 61 euros) a month [27].

These multiple uses of products derived from *B. aethiopum* have put a strong anthropogenic pressure on the species, thus contributing to both fragmentations of its populations and their poor natural regeneration [27, 29–32]. Further fragmentation of the species’ habitat has been observed as a result of land clearing for agriculture or urban development [32–34]. As illustrated through similar examples in the literature [35, 36], such phenomena may lead to restricted gene flow and ultimately, to loss of genetic diversity among *B. aethiopum* populations. A sustainable management policy for *B. aethiopum* populations is therefore urgently needed and acquiring information on the genetic diversity of the species and population structure is a major step towards defining sustainable management actions. At the time of writing the present article, only a few chloroplast sequences are publicly available for *B. aethiopum* through NCBI (https://www.ncbi.nlm.nih.gov/search/all/?term=borassus%20aethiopum). By contrast, abundant molecular resources, including genome assemblies or drafts, are available for model palm species such as *Elaeis guineensis* Jacq [37]., *Phoenix dactylifera* L. [38–40] and *Cocos nucifera* L. [41, 42]. In each of these three palm species, large numbers of SSR markers have been identified and for a fraction of them, cross-species and cross-genera transferability tests among species belonging to the Palmaceae family have been performed [43–49]. In several instances [44–47, 49] these tests included samples from *Borassus flabellifer*, the Asian relative of *B. aethiopum*.

The primary objective of the present study is to generate the first set of genetic data on *Borassus aethiopum,* as a first step towards improving the management of this species through a better knowledge of its diversity. In order to achieve this, we first describe attempts to use SSR markers identified in these other palm species. Then, we describe the low-coverage sequencing of the *B. aethiopum* genome with the aim of developing the first set of specific SSR markers targeting this species. Finally, we used the novel SSR markers to assess the genetic diversity and population structure of *B. aethiopum* samples collected across the three different climatic regions of Benin, a country that was most readily accessible to us for sample collection, as an important first step towards more comprehensive studies spanning the West African sub-region.

## Results

### Assessment of palm SSR marker transferability to *Borassus aethiopum* and evaluation of their capacity for characterizing genetic diversity

Of the 80 microsatellite markers selected from the three model palm species *Elaeis guineensis*, *Phoenix dactylifera* and *Cocos nucifera* and tested for amplification on *B. aethiopum* DNA, 18 (22.5%) generate amplification products (Table 1). No amplification is observed using the 11 *C. nucifera* markers, whereas 7 (15.9%) and 11 (44%) of the *P. dactylifera* and *E. guineensis* markers, respectively, show a successful amplification. None of the amplification products generated with *P. dactylifera* primers display genetic polymorphism in our *B. aethiopum* test panel. Among *E. guineensis*-derived SSR markers however, two, namely ESSR566 and ESSR652, display polymorphism. However, it must be noted that depending on the DNA sample the ESSR566 primer pair generates a variable number of amplicons with distinct sizes, which may be an indication that more than one locus is targeted.

**Table 1:** Summary of SSR markers transferability assessment

Species of origin	Number of SSR markers tested	Number of successful amplifications (% of markers)	Number of polymorphic amplicons (% of amplifications)
*Cocos nucifera*	11	0 (0)	0 (0)
*Phoenix dactylifera*	44	7 (15.9)	0 (0)
*Elaeis guineensis*	25	11 (44.0)	2 (18.2)
Total	**80**	**18 (22.5)**	**2 (11.1)**

Overall, during this phase of the study we detect polymorphism in our *B. aethiopum* test panel with only 2 (11.1% of successfully amplified markers, 2.5% of total) of the palm SSR primer pairs assayed. Only one of these markers, namely ESSR652, enables unambiguous detection of microsatellite locus polymorphism in *B. aethiopum*, and might therefore be used for studying genetic diversity in this species.

### De novo identification of microsatellite sequences in the *B. aethiopum* genome and assessment of potential SSR markers

In order to enable a more precise evaluation of genetic diversity in *B. aethiopum*, we developed specific *B. aethiopum* markers from de novo sequencing data. A total of 23,281,354 raw reads with an average length of 250 bp have been generated from one MiSeq run. Raw sequence reads have been trimmed resulting in 21,636,172 cleaned-up reads, yielding 493,636 high-quality reads after filtering (Q > 30) from which 216,475 contigs have been assembled.

From the contigs, the QDD software identifies a total of 1618 microsatellite loci (Additional file 1), of which 1327 (82.01%) are perfect (i.e. repeat size 4 bp or smaller and repeat number 10–20). Among the perfect microsatellite loci, 83.86% are composed of di-nucleotidic repeat units, 13.06% of tri-nucleotidic units, 2.39% of tetra-nucleotidic repeats and 0.67% of repeats with five nucleotides and over. From these, we selected SSR markers composed of di- (AG) or tri- nucleotide repeats, using the following criteria for specific amplification of easily scorable bands: primer lengths ranging from 18 to 22 bp, annealing temperatures 55–60 °C, and predicted amplicon sizes 90–200 bp.

The characteristics of the 57 selected primer pairs and the results of the test amplifications are presented in Table 2. Successful amplification of *B. aethiopum* DNA is obtained for 54 (94.7%) primer pairs and of these, 34 (60.0% of amplifying couples) show no polymorphism. The remaining 20 primer pairs enable the amplification of polymorphic products, however nine of them yield complex, ambiguous amplification profiles that prevent their use for reliable detection of genetic variation. As a result, 11 putative *B. aethiopum* SSR markers (representing 20.4% of primer pairs associated with successful amplification and 55.0% of those detecting polymorphic products in our study) are both polymorphic and unambiguously mono-locus in our amplification test panel and may therefore be used for further analyses.

**Table 2:** List of selected primer pairs targeting putative *B. aethiopum* microsatellite loci and assessment of their polymorphism detection ability

Locus name	Repeat motif	Primer sequences (5′-3′ orientation)	Expected amplicon size (bp)	Amplification product
**MBo01**	[AGG]_7_	CCTATCCTTCCATCCCGATCG	90	complex, polymorphic
		TTGCCGTGAATCAGCCTCAA		
**MBo02**	[ATC]_7_	GGGAGAACAAGGATAACAGCAG	115	single locus, monomorphic
		TCCATTTCATCACTAGCTCGGT		
**MBo03**	[AGG]_7_	CTCCGAGCCCTAGCAACTTT	131	single locus, monomorphic
		TCTGGATGACGAAACCTTCACA		
**MBo04**	[ACC]_7_	GATGTGGCCGCTCTGATCTC	192	single locus, monomorphic
		ACATGCTGGCAAGGTATTCT		
**MBo05**	[AAG]_7_	GTCCTAGCACGCTGGCATTA	202	single locus, monomorphic
		TGGGTTGCCAATGAACCCTT		
**MBo06**	[ATC]_7_	TGGCCATTCAACTGCTTCAC	202	single locus, monomorphic
		GAATCTAGCACCAGCAAACCC		
**MBo07**	[AAG]_7_	GGCACTGGAGTCCACATCAA	239	single locus, monomorphic
		TCCTTCTGTACTGGCATCTCT		
**MBo08**	[AGG]_8_	TGATTGTTTCCTCTTCCCTCCT	90	single locus, monomorphic
		TTAATGAGCCGAAGAGGAGCC		
**MBo09**	[AGG]_8_	TCCCTCACTCCCATCCTCTC	163	single locus, monomorphic
		ACTCCACTCCTTCCCTCATACA		
**MBo10**	[AAC]_8_	GTTAAAGACGCAGGGCTGGA	166	single locus, monomorphic
		CCCACTTAGTGAGATAAGACTTGA		
**MBo11**	[ATC]_8_	GCATCACATGGTTTCAGGCT	219	single locus, monomorphic
		GCTCAACCATCGGCAGTGTA		
**MBo12**	[ATC]_9_	GGAGGAAAGGTTGCCCTAGAA	102	single locus, monomorphic
		TCTCAACCTGATGTCATTGCA		
**MBo13**	[AAG]_9_	CAGGTTGCATCGGCCCATT	103	complex, polymorphic
		GGAGCCTAATGCACCCAGAG		
**MBo14**	[AAC]_9_	ATGGCCGATCCCACTTAGTG	117	single locus, monomorphic
		GAGAGAACGGCAATAATTTATGCA		
**MBo15**	[AAG]_10_	GCTGAAGAGGATGAAGAAGAAGC	92	complex, monomorphic
		TCATCATCTCCCTCTCCTTCT		
**MBo16**	[AGG]_10_	CAGCACTGGCCTCACAGC	118	single locus, monomorphic
		CCGTCGATCAGTTGTTGGAGA		
**MBo17**	[ATC]_10_	ACACAATGACCTTTCGCTGA	124	single locus, monomorphic
		CCAAACAGGACCTTATGCCA		
**MBo18**	[AAG]_10_	ACATCCTCTCCTTCATCTCCTT	187	complex, polymorphic
		GTTCCTACAATGCTTGGCGC		
**MBo19**	[AAG]_10_	TGCTATCACCCAATATCTAGGCT	202	single locus, monomorphic
		ACAGTCAACAACTACCATACTGC		
**MBo20**	[AAG]_10_	TGTGGTTAAAGCAATGGAAGCA	229	single locus, monomorphic
		GCCGAACTCCTACTCTCATACG		
**MBo21**	[AAG]_11_	ACAACAGAAGATCAGTATACGTTCT	171	single locus, monomorphic
		TTGAGGAATCATGCTTGTCAGT		
**MBo22**	[AAG]_14_	AGAAGAATTCGGTTAGGTCACAA	108	single locus, monomorphic
		AGATAACATGGGTAAGAATTGCCT		
**MBo23**	[AAT]_5_	TGAGTTCTTGTCTTGTCTTCGT	100	single locus, monomorphic
		GGTTTGGGACACCCTTCAGG		
**MBo24**	[AAT]_9_	AAAGTCATGTCTGGGTGATGAA	90	single locus, monomorphic
		ATGATGAGCACAGCTACAACTCT		
**MBo25**	[AAT]_6_	TCTTCAGGTGACAAGCAACA	96	single locus, monomorphic
		CCTGGGCATGGAGATAGCAT		
**MBo26**	[AAT]_7_	CCATAGGCCAGCCCACTATA	134	single locus, monomorphic
		ACCCTTTCTTCTTCCTCATTTGT		
**MBo27**	[AAT]_7_	TCTCTATTGCTTGGTGATCCC	103	single locus, monomorphic
		TCCAACAAGGGATGGTTATCATG		
**MBo28**	[AAT]_8_	GCCTTGAGAGTGGAAGAGGC	205	single locus, monomorphic
		TCTCTTCTTTGCGCCCTCAT		
**MBo29**	[AAT]_16_	AGACATGTAGAGGTGGGACT	211	single locus, monomorphic
		TCTGTATGAGAGACGTGTTACAGT		
**MBo30**	[AAT]_8_	TGACCATAACAAGCTACCAGGT	146	single locus, monomorphic
		GGTGGAAGCTATTGATATTGCATGT		
**MBo31**	[AAT]_10_	TGACAATGATGCATGCGATAACA	187	single locus, monomorphic
		GCATCACCCATGTCCTTTAGC		
**MBo32**	[AAT]_10_	TCCGAGGGCAGTATTTGTCG	117	single locus, monomorphic
		CACTATTTCGGAAACCTAAGCCC		
**MBo33**	[AAT]_17_	GCACACTTTGTATCCGACGC	147	single locus, monomorphic
		CAGGGATAGTAACCGTCAGGG		
MBo34*	[AG]_28_	GTGGCACCTCTGCGGTTT	192	single locus, polymorphic
		CGAGATGGAAGCACCTGGAG		
MBo35*	[AG]_24_	AGCATGCTTTCTGCTTCATGTG	137	single locus, polymorphic
		CCTTTCCCTGACTGCATTGC		
**MBo36**	[AG]_23_	TCGGAAGTCGAATGTGGCAG	180	no amplification
		TCGGAAGAGTGGTCAATCATGG		
**MBo37**	[AG]_23_	GCTCTACTCCCAGAGACGGA	142	complex, polymorphic
		AACAGTCGACGGAATGCTCA		
MBo38*	[AG]_20_	AGTCCTCACTGCTGGTGGTA	130	single locus, polymorphic
		TCCTTGAATAGTCCATCTTGCA		
**MBo39**	[AG]_19_	AACGCAGGTTAAGAGGCTCC	168	complex, monomorphic
		CCTCCTGGTGCAACCCTTAC		
**MBo40**	[AG]_19_	TGTGGAGTGTGAGTCGATGG	193	complex, polymorphic
		GGCTGCATAATCTCATCACGC		
MBo41*	[AG]_18_	TTCTCCACCAGCCTCACAAC	184	single locus, polymorphic
		ATACGGCCCATCAACCCTTC		
**MBo42**	[AG]_18_	CCTGGTGGTACATGTGGTCA	136	complex, polymorphic
		TGTGGCACATTCATTTCTGAAGG		
**MBo43**	[AG]_18_	AGTTTGTTCTGTGTGTTGTCAC	137	no amplification
		GCACACATCTTGCTTTGAAGAC		
**MBo44**	[AG]_17_	AACACACTTTAAATCGACTTCTTCA	193	complex, polymorphic
		CACGGCTGCCATGTGAGG		
**MBo45**	[AG]_17_	TAGATCGGAAGTCAGGCCC	193	no amplification
		AGAGAAGTGGGAGGAGAGGTC		
**MBo46**	[AG]_17_	GCCGATATTAGCTTCTTCTTGGC	154	single locus, monomorphic
		GCCTTGTTGATCCCGTTTCAC		
**MBo47**	[AG]_16_	GGCACCTGACGCCTCTTT	188	single locus, monomorphic
		TCACTTCGACTCAATTGTATCCAT		
**MBo48**	[AG]_16_	AGGACAAAGAGATGAGAAGCCT	92	complex, polymorphic
		ACCAATTCCCAGTTAGTTGACCA		
MBo49*	[AG]_16_	CATCACCCATTCTCTCTGCCT	141	single locus, polymorphic
		GAGAAACCATCCGCACCTCA		
MBo50*	[AG]_15_	AGAAGTCATCTTGAGGGCCC	150	single locus, polymorphic
		TTGCTAGAATGATACACAAATTGCT		
MBo51*	[AG]_15_	TGTGCTATTTGTTGGGAATGCA	191	single locus, polymorphic
		GCAAGCTCATGTTCTAGTTTCAAGT		
MBo52*	[AG]_15_	ACACATCCTACATGAATAGACCTCC	122	single locus, polymorphic
		TCTTGTCATAGCCTAGATTCCCT		
**MBo53**	[AG]_15_	AGGTTTAAGGGTTTGGGTTAGGG	131	single locus, monomorphic
		GGTGGAGTAAGTTTGAGGGTCA		
MBo54*	[AG]_11_NNN[AG]_15_	CATATGCTGATACAAGAGAGAGGG	124	single locus, polymorphic
		ACCTTATAAGCAGGATCCAGACA		
**MBo55**	[AG]_15_	TGGAATCAACCTTGGGTCTACA	198	complex, polymorphic
		TCGTCGGTCTTCTAGCCACT		
MBo56*	[AG]_15_	ACCAAGATCAAGCACGAGGA	103	single locus, polymorphic
		AGGATCACCCTTTCTTTCTTTCT		
MBo57*	[AG]_15_	GGGTTCAATCCTGATGAGAGCA	136	single locus, polymorphic
		ACCGTTCGATCAACCATGGT		

Loci for which single-locus SSR polymorphism has been detected within our test panel of seven *B. aethiopum* individuals are signaled by an asterisk (*)

Conventionally, microsatellite motifs are displayed under the form [N_1_N_2_]_x_ or [N_1_N_2_N_3_]_x_ for dinucleotide and trinucleotide loci, respectively, where N_1_, N_2_ and N_3_ represent nucleotides included in the elementary unit of the motif and x is the number of unit repetitions. Expected amplicon size is as predicted by QDD

### Microsatellite-based characterization of genetic variation of *B. aethiopum* in Benin

**Fig. 1:**
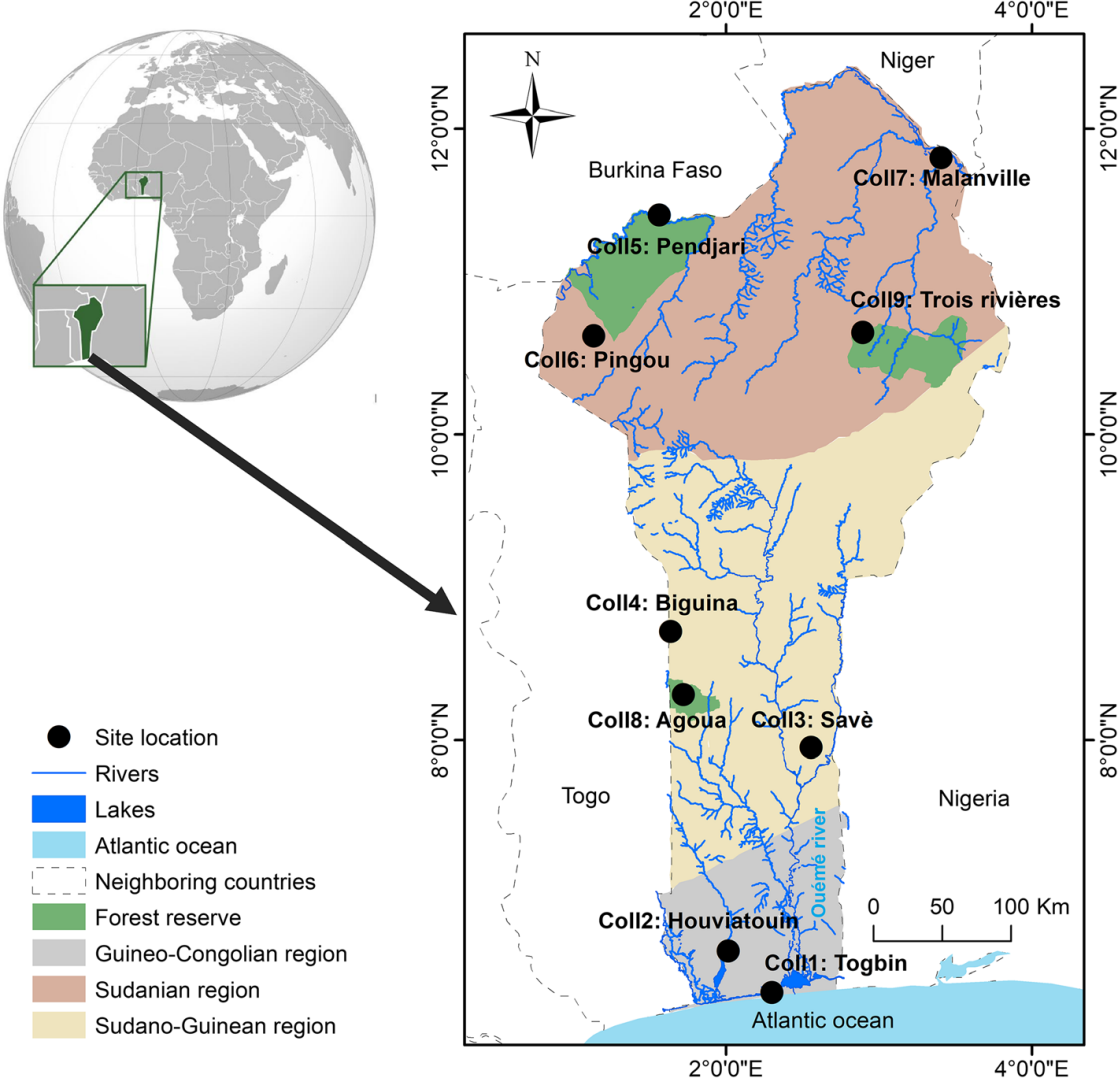
Sampling locations of the Beninese *B. aethiopum* used in this study

The newly identified set of 11 *B. aethiopum*-specific SSR markers has been used for the characterization of genetic diversity in our full panel of 180 individual samples from nine locations distributed across Benin (Fig. 1).

Map generated from publicly available resources of the Institut Géographique National du Bénin (IGN; https://geobenin.bj/) and the “Major Rivers of the World” dataset from the World Bank Data Catalog (https://datacatalog.worldbank.org/dataset/major-rivers-world; Creative Commons Attribution 4.0 International license), using the ArcGIS software by ESRI (www.esri.com).

As shown in Table 3, among our sample set the number of alleles per microsatellite locus ranges from 2 for marker Mbo41 to 6 for markers Mbo34, Mbo35, and Mbo50, with an average value of 4.27, whereas expected heterozygosity (He) values range from 0.031 (marker Mbo56) to 0.571 (marker Mbo35). Using these markers, the analysis of genetic diversity (Table 4) shows that the number of polymorphic markers detected at the microsatellite loci investigated ranges from 8 (sites of Togbin and Malanville) to 10 (Savè, Agoua, Pendjari, Pingou and Trois Rivières), with a mean value of 9 ± 0.865. With the exception of Savè, Hounviatouin and Malanville, 1 to 3 private alleles of the targeted microsatellite loci are observed in most sampling locations. Regarding the genetic parameters, the number of effective alleles (Ne) ranges from 1.447 to 2.069 with an average number of 1.761. He values range from 0.263 (Hounviatouin) to 0.451 (Savè) with an average value of 0.354 whereas the observed heterozygosity (Ho) varied from 0.234 (Togbin) to 0.405 (Pingou) with an average value of 0.335. Negative values of Fixation index (F) are obtained for Pingou, Malanville and Trois Rivières whereas positive F values, indicating a deficit of heterozygosity, are observed in all other sites investigated.

**Table 3:** Characteristics of 11 newly identified polymorphic microsatellites markers used for genetic diversity analysis of *B. aethiopum*

Locus name	Number of alleles scored/locus	Expected Heterozygosity (He)	Observed Heterozygosity (Ho)
**Mbo34**	6	0.520	0.383
**Mbo35**	6	0.571	0.522
**Mbo38**	5	0.458	0.513
**Mbo41**	2	0.343	0.356
**Mbo49**	4	0.167	0.146
**Mbo50**	6	0.548	0.542
**Mbo51**	3	0.320	0.304
**Mbo52**	3	0.201	0.232
**Mbo54**	4	0.26	0.435
**Mbo56**	3	0.031	0.034
**Mbo57**	5	0.296	0.263

**Table 4:** Mean diversity parameters for each of the nine *B. aethiopum* sampling sites

Geo-climatic region	Site	Nb of polymorphic markers	Na	Ne	Nb of private alleles	Allelic richness	Ho	He	F
Guineo-Congolian (South)	*Togbin*	8	2.273	1.584	3	2.08	0.234 **±** 0.063	0.288 **±** 0.073	0.145 **±** 0.066
	*Hounviatouin*	9	2.182	1.447	0	2.1	0.272 **±** 0.066	0.263 **±** 0.054	0.007 **±** 0.094
Sudano-Guinean (Center)	*Savè*	10	2.909	2.069	0	2.72	0.384 **±** 0.075	0.451 **±** 0.066	0.134 **±** 0.088
	*Biguina*	9	2.364	1.770	2	2.21	0.345 **±** 0.069	0.374 **±** 0.070	0.064 **±** 0.062
	*Agoua*	10	2.273	1.722	1	2.16	0.329 **±** 0.063	0.358 **±** 0.066	0.059 **±** 0.071
Sudanian (North)	*Pendjari*	10	2.818	1.900	3	2.49	0.368 **±** 0.070	0.396 **±** 0.071	0.055 **±** 0.064
	*Pingou*	10	2.364	1.906	1	2.29	0.405 **±** 0.072	0.390 **±** 0.073	0.063 **±** 0.049
	*Malanville*	8	2.455	1.627	0	2.2	0.302 **±** 0.072	0.303 **±** 0.074	0.020 **±** 0.051
	*Trois Rivières*	10	2.545	1.822	2	2.43	0.373 **±** 0.074	0.360 **±** 0.073	0.055 **±** 0.039
	**Overall mean**	**9 ± 0.865**	**2.465 ± 0.103**	**1.761 ± 0.065**			**0.335 ± 0.023**	**0.354 ± 0.023**	**0.035 ± 0.022**

Values are provided **±** standard deviation wherever applicable

*Na* average number of different alleles, *Ne* effective number of alleles, *Ho* Observed Heterozygosity, *He* Expected Heterozygosity, *F* Fixation index

### Population structure of *B. aethiopum* in Benin

Nei’s genetic distance among locations (Table 5) ranges from 0.073, as observed between Togbin and Hounviatouin (Guineo-Congolian region), to 0.577 between Togbin (Guineo-Congolian region) and Trois Rivières (Sudanian region). Overall, genetic distances between *B. aethiopum* sampling locations are lowest within the same region, with the lowest genetic distances among the sites of Pendjari, Pingou, and Trois Rivières which are all located in the Northern part of the country. One interesting exception is the Central (Guineo-Sudanian) region of Benin, where we find that the most genetically distant location from Savè is the one from the Agoua forest reserve (0.339). Surprisingly, Savè displays its highest genetic identity value when compared to the other two collection sites located within protected areas, namely Pendjari (0.870) and Trois Rivières (0.882) which are both located in the Sudanian region. This is an unexpected finding considering the geographic distances involved.

**Table 5:** Pairwise location matrix of Nei’s genetic distance and genetic identity values

	Togbin	Hounviatouin	Savè	Biguina	Agoua	Pendjari	Pingou	Malanville	Trois Rivières
Togbin	**–**	0.073	0.477	0.253	0.337	0.517	0.494	0.487	0.577
Hounviatouin	0.929	–	0.419	0.110	0.215	0.435	0.317	0.375	0.535
Savè	0.621	0.658	–	0.270	0.339	0.140	0.265	0.238	0.126
Biguina	0.776	0.896	0.763	–	0.152	0.241	0.161	0.186	0.316
Agoua	0.714	0.806	0.713	0.859	–	0.408	0.304	0.359	0.490
Pendjari	0.596	0.647	0.870	0.786	0.665	–	0.167	0.108	0.103
Pingou	0.610	0.728	0.767	0.851	0.738	0.846	–	0.174	0.175
Malanville	0.614	0.688	0.788	0.831	0.699	0.898	0.841	–	0.145
Trois Rivières	0.561	0.585	0.882	0.729	0.613	0.902	0.840	0.865	–

Above the diagonal: Nei’s genetic distance; below: genetic identity

A similar structure of genetic distances emerges from the analysis of pairwise location genetic differentiation (Fst) (Table 6), suggesting genetic differentiation according to geographic distances between collection sites, with the notable exception of the lower genetic differentiation between samples from Savè and those from either one of the forest reserves in the Northern region, namely Pendjari and Trois Rivières.

**Table 6:** Pairwise sampling locations Fst value

	Togbin	Hounviatouin	Savè	Biguina	Agoua	Pendjari	Pingou	Malanville	Trois Rivières
Togbin	0.000								
Hounviatouin	0.072	0.000							
Savè	0.233	0.221	0.000						
Biguina	0.168	0.086	0.145	0.000					
Agoua	0.215	0.153	0.157	0.105	0.000				
Pendjari	0.247	0.212	0.077	0.120	0.188	0.000			
Pingou	0.252	0.181	0.138	0.103	0.169	0.100	0.000		
Malanville	0.301	0.246	0.149	0.121	0.197	0.072	0.119	0.000	
Trois Rivières	0.285	0.279	0.076	0.178	0.224	0.073	0.104	0.107	0.000

**Fig. 2:**
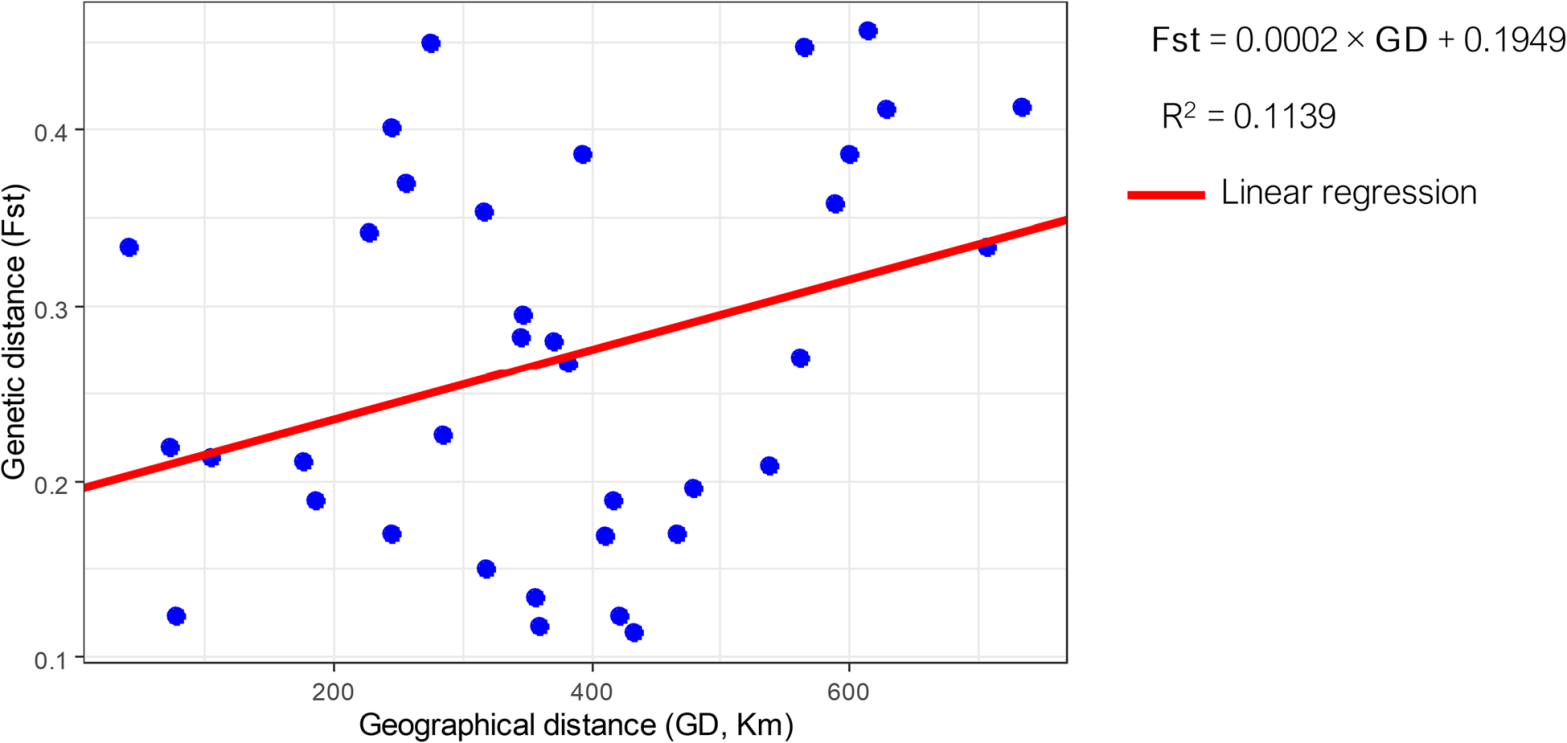
Correlation between pairwise Fst vs. pairwise geographical distance

In order to assess the strength of the relationship between genetic and geographic distances, we plotted them as a linear regression and performed the Mantel permutation test. As shown in Fig. 2, the positive correlation between both variables is weak, but significant (*R*^*2*^ = 0.1139, *P* = 0.040).

The results of the non-hierarchical AMOVA (Table 7) show that within-site variation underlies the major part (53%) of total variance, whereas among-sites and among-regions variations explain genetic variance to a similar extent (23 and 24%, respectively). Accordingly, the average Number of migrants between collection sites (Nm = 1.019) is low, indicating very limited gene flow.

**Table 7:** AMOVA results

Analysis	Scale	df	SS	MS	Est. var.	% Total variance	*P* value
Non-hierarchical AMOVA	Among regions	2	309.407	154.704	1.944	24%	< 0.001
	Among sites	6	254.302	42.384	1.903	23%	< 0.001
	Within sites	171	739.100	4.322	4.322	53%	< 0.001
	*Total*	*179*	*1302.809*		*8.169*	*100%*	
Hierarchical AMOVA, K = 2	Among regions	1	66.765	66.765	0.205	7%	
	Among sites	7	212.421	30.346	0.703	24%	
	Among individuals	171	379.675	2.220	0.195	7%	
	Within individuals	180	329.500	1.831	1.831	62%	
	*Total*	*359*	*988.361*		*2.933*	*100%*	
	***F-statistics***	***Value***	***P-value***				
	Fst	0.310	0.010				
	Fis	0.096	0.010				
	Fit	0.376	0.010				
Hierarchical AMOVA, K = 3	Among regions	2	152.676	76.338	0.478	16%	
	Among sites	6	126.510	21.085	0.472	16%	
	Among individuals	171	379.675	2.220	0.195	7%	
	Within individuals	180	329.500	1.831	1.831	62%	
	*Total*	*359*	*988.361*		*2.975*	*100%*	
	***F-statistics***	***Value***	***P-value***				
	Fst	0.319	0.010				
	Fis	0.096	0.010				
	Fit	0.385	0.010				

*df*  degree of freedom, *SS*  sum of squares, *MS* mean squares, *Est. var.*  estimated variance, *Fst* inter-sites genetic differentiation, *Fis* genetic differentiation of individuals within sites, *Fit* differentiation of individuals from the total

Hierarchical analyses performed with K = 2 and K = 3, respectively, yield an identical proportion of genetic variation at the within-individual level (62% of total; Table 7). Analysis using K = 3 allows for a balanced representation of variation between the among-regions and among-sites scales (16% of total variance for each), whereas among-regions variation is not as well accounted for under K = 2 (7% of total variance, vs. 24% for among-sites variation).

**Fig. 3:**
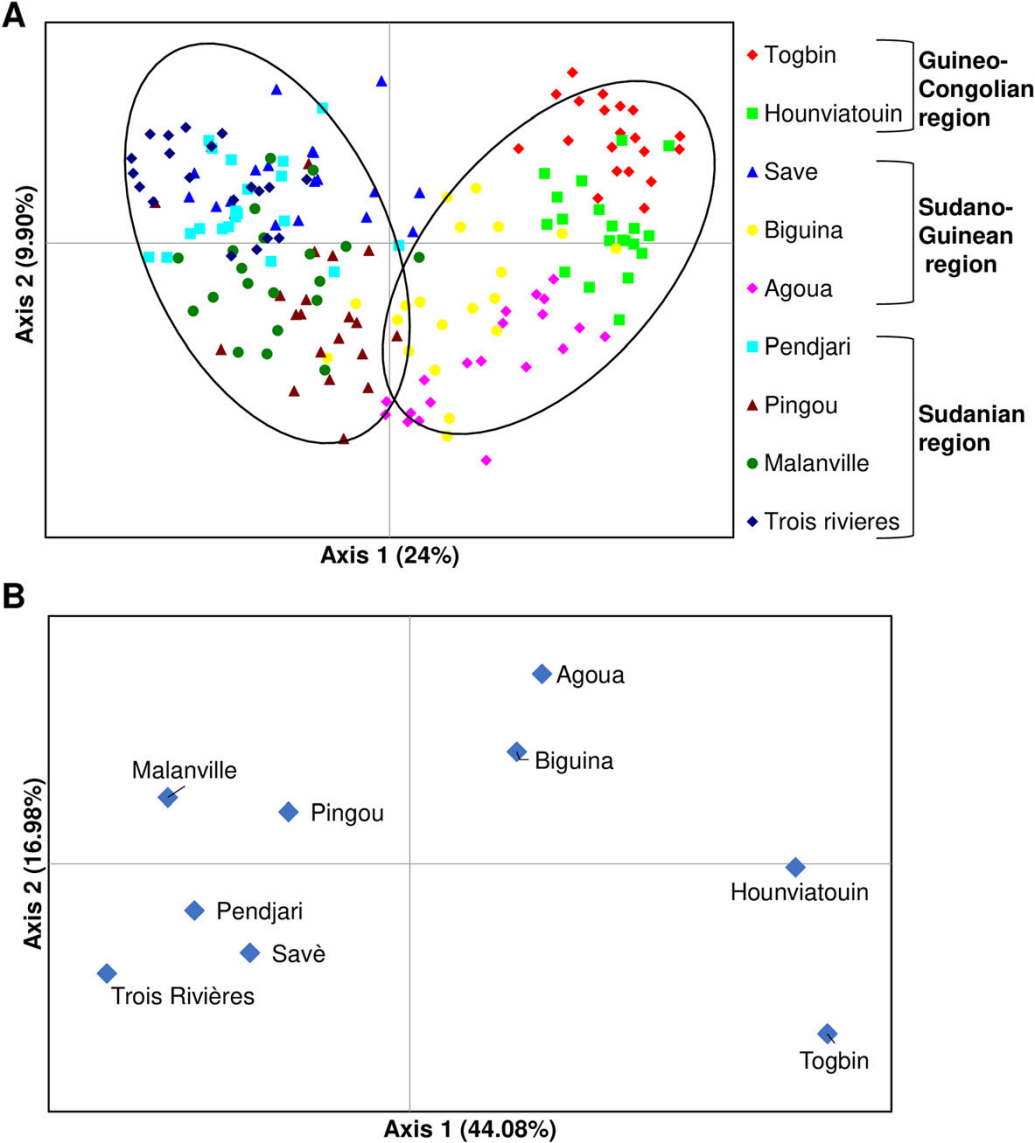
Principal Coordinates Analysis (PCoA). **a** PCoA analysis of individual *B. aethiopum* samples. **b** Sampling locations-based PCoA

The Principal Coordinates Analysis (PCoA) of 180 *B. aethiopum* samples (Fig. 3a) shows that the first axis (accounting for 24% of total variation out of a sum of 33.90 for axes 1 and 2) roughly separates individual samples in two main groups, a result that is in agreement with the analysis of genetic distances. The sampling locations-based PCoA (Fig. 3b) confirms the genetic separation along the first axis (accounting for 44.08% of total variation over a total of 61.06% for the sum for axes 1 and 2) between sites from the Guineo-Congolian (Southern) region, plus the sites of Agoua and Biguina (Center) vs. sites from the Sudanian (Northern) region, plus the site of Savè (Center). Although the distinction is not as clearly marked, the second axis (accounting for 16.98% of total variation) further allows to distinguish two subgroups within the first group, corresponding to sites belonging to the Southern region and to those from the Central one, respectively.

**Fig. 4:**
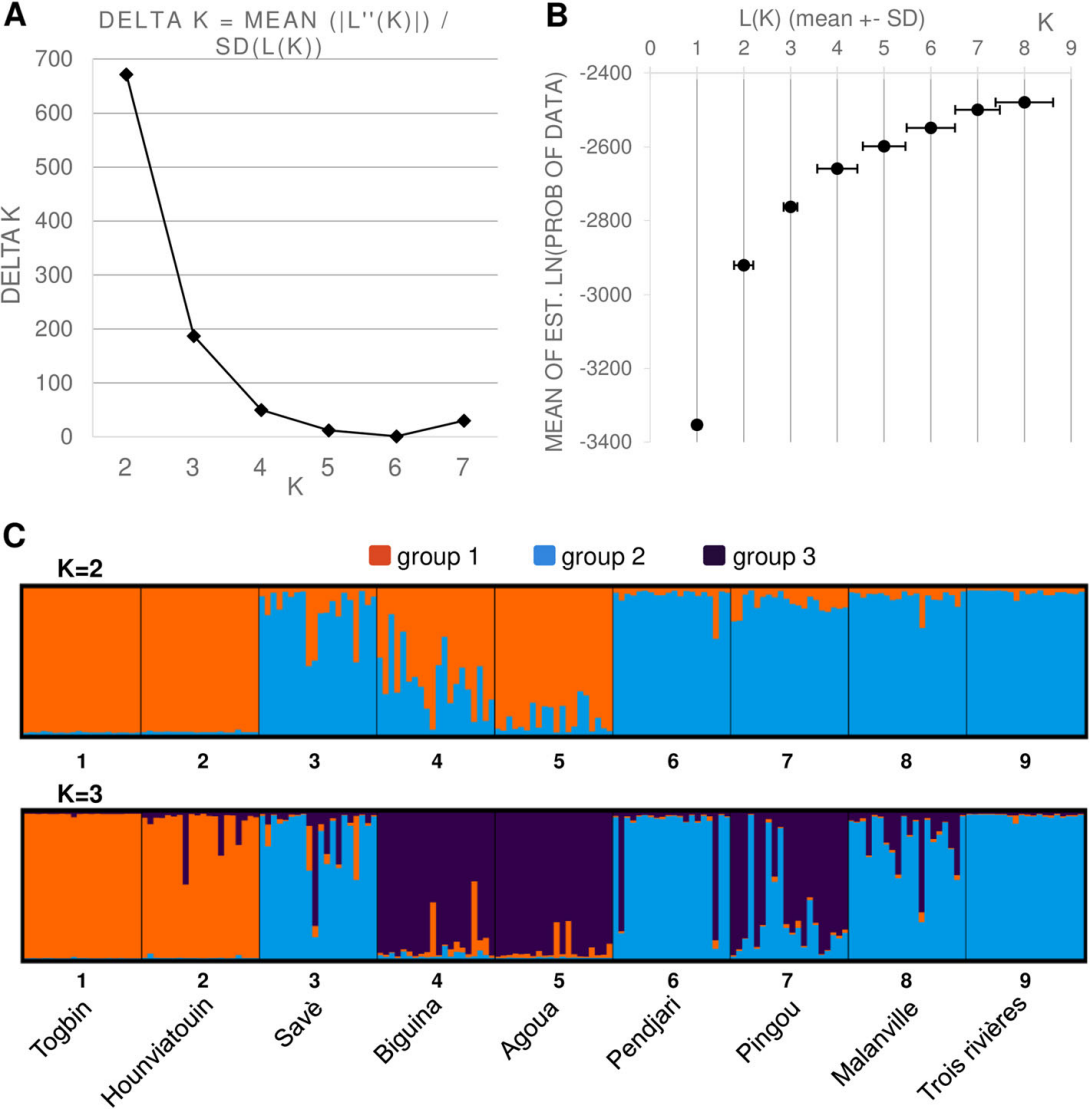
Bayesian cluster analysis. **a** Determination of the optimal value of K from Structure Harvester. **b** Evanno plot. **c** Bar plot representations of Bayesian STRUCTURE analysis of Beninese *B. aethiopum* samples with K = 2 (top) or with K = 3 (bottom) generated with CLUMPAK. Sampling sites are displayed along the horizontal axis

Likewise, the Bayesian analysis of our data indicates an optimal value of K = 2 for the clustering of the samples into two groups (Fig. 4a and b): one group that includes samples from Togbin and Hounviatouin in the Southern part of the country, as well as most samples from Biguina and Agoua at the Western (Togolese) border of the Center region; and one group composed of the majority of samples collected in Savè (Eastern part of the Center region) and from the Northern locations of Pendjari, Pingou, Malanville, and Trois Rivières. Since there is a possibility that the ΔK method used for estimating K leads to over- or under-estimated values [50], clustering with higher values of K have also been tested. As is apparent in Fig. 4b, for values of K = 4 and above standard deviations increase considerably, therefore we present results for both K = 2 and K = 3 (Fig. 4c; see also Additional Figure 4 for the summary of the complete analyses with K = 1 to K = 10). As previously observed with the location-based PCoA, under K = 3 further clustering emerges within the first group, involving samples from Togbin and Hounviatouin (South) and those from Biguina and Agoua (Center), respectively.

**Fig. 5:**
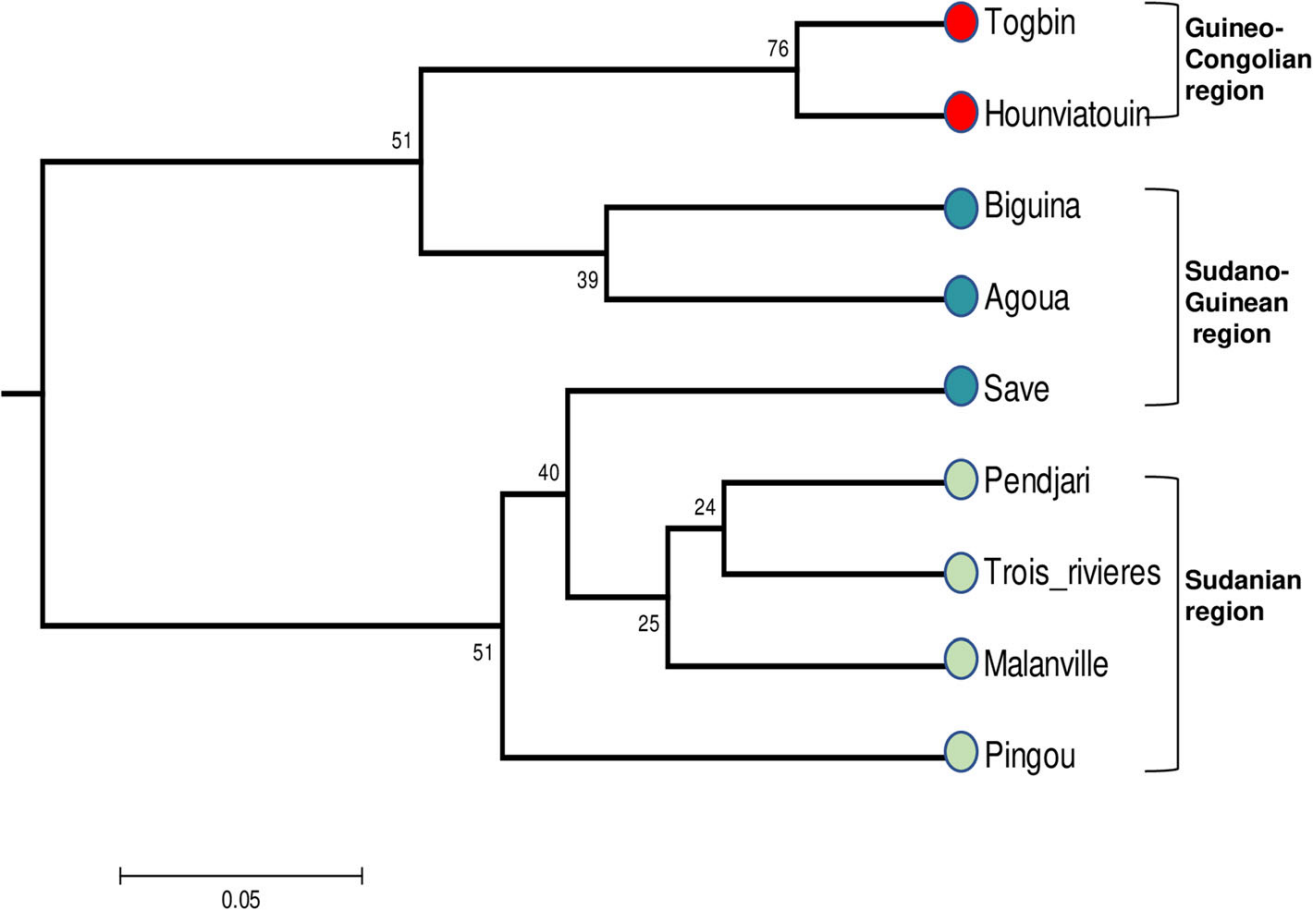
UPGMA dendrogram based on genetic distances between Beninese *B. aethiopum* sampling locations. Bootstrap values supporting each branch are indicated on the nodes

The Unweighted pair-group method with arithmetic mean (UPGMA) tree constructed from our data (Fig. 5) distinguishes two main groups matching the ones defined through the Bayesian analysis with K = 2, and which are supported by bootstrap values above 50. Within each of these groups, subgroups corresponding to those observed with K = 3 clustering and that globally match geo-climatic regions (Savè excepted) can further be defined. However, in this case most bootstrap values attached to these secondary branches are not significant.

## Discussion

In flowering plant, the efficiency of cross-species transfer of SSR markers is highly variable among taxa, especially when important differences in genome complexity exist between the marker source and the target [51]. Nevertheless, this method has been used successfully for accelerating the analysis of genetic diversity in many plant species, including palms [11, 52–54]. In the present study, we find that the transferability rate of microsatellite markers developed in other palms genera to *Borassus aethiopum*, i.e. their ability to successfully amplify genomic DNA from the latter species, is very low. Indeed, among the 80 primer pairs designed on either *Elaeis guineensis*, *Phoenix dactylifera* or *Cocos nucifera*, we observe that only 18 (22.5%) produce amplicons from *B. aethiopum*. This percentage is very low when compared to both the inter-species and inter-genera transferability rates that have been found in similar studies targeting other palm species: from 17 to 93% in a panel of 32 palm species [49], 75% from *E. oleifera* to *E. guineensis* [54], 86% between the wooly jelly palm (*Butia eriospatha* Mart.) and related species *Butia catarinensis* [55] and up to 100% in the licuri palm (*Syagrus coronate* Mart) [56]. When considering other plant families, our transferability rate is also markedly lower than both the average rate of 50% found by Peakall et al. [57] within the *Glycine* genus and among Legumes genera, and the overall rate of 35.2% calculated by Rossetto [58] for within-family transferability among Gymnosperms and Angiosperms. The low transferability rate in our study might be explained in part by the fact that we used markers originating from genomic sequences. Indeed, as pointed out by Fan et al. [1], such markers have a lower transferability rate when compared to Expressed Sequence Tags (ESTs)-derived microsatellites due to the higher inter-species sequence variability within non-coding vs. coding sequences. Similarly, it is plausible that differences in genome size and complexity among palm species and genera account for our difficulty to identify palm SSR markers that successfully amplify in *B. aethiopum*. As a matter of fact, the size of the *B. aethiopum* genome, as determined by flow cytometry (1C = 7.73 Gb; Jaume Pellicer, unpublished data), is 3.2 to 11.5 times larger than those of the microsatellite source species used in the present study: *P. dactylifera* genome is estimated to be 671 Mb [39] whereas the *E. guineensis* genome is 1.8–1.9 Gb [37, 59] and *C. nucifera* genome is 2.42 Gb [42]. It is possible that these differences in genome sizes among related diploid plant species rely on differences in transposable element (TE) content, which in turn might have induced structural alterations throughout the genome through indels, copy number variations and recombinations [60, 61]. The illustration of such a mechanism working at the intra-genus level has been provided by cultivated rice species *Oryza sativa* L. and its wild relative *Oryza australiensis* [60]. Ultimately, TE-induced structural variations may have a negative effect on the cross-species amplification ability of some of the SSR primers. Indeed, in a recent study Xiao et al. [49] showed that over 70% of the conserved microsatellite loci between *E. guineensis* and *P. dactylifera* are located within genic regions of the genome with low TE content, and which are therefore less likely to be submitted to TE-dependent structural variations. More generally, gaining a better understanding of genome structures within the *Borassus* genus could also help reconcile our results with previous published reports of successful transfer of SSR markers developed from other palm sources to *Borassus flabellifer* (see references cited in Table 8, Methods section). Indeed, since the genome size of *B. flabellifer* (7.58 Gb; Jaume Pellicer, unpublished data) is only marginally smaller than that of *B. aethiopum*, significant differences in genome composition may be underlying the lack of SSR transferability between both species.

**Table 8:** Characteristics of the palm SSR markers tested for transferability to *B. aethiopum*

Marker name	Primer sequences (5′-3′ orientation)	T_**a**_ (°C)	Source palm species	Successful transfer to other palm species	References
mEgCIR0230	CCCTGGCCCCGTTTTTC	57.0	*E. guineensis*	*E. oleifera* *Syragus* sp*.* *C. nucifera* *P. roebelinii* *P. canariensis* *P. reclinata*	[62]
	AGCGCTATATGTGATTCTAA				
mEgCIR0326	GCTAACCACAGGCAAAAACA	59.0			
	AAGCCGCACTAACATACACATC				
mEgCIR0465	TCCCCCACGACCCATTC	63.1			
	GGCAGGAGAGGCAGCATTC				
mEgCIR0476	TTCCTCGGCCCCTTCTC	61.6			
	TCGCCGACCTTCCACTG				
EgCSSR-5781	TTCACGCTACTGATGGTTGG	59.4	*E. guineensis*	*B. flabellifer*	[49]
	TCGATCCCTTCTCTGGAAAC				
EgCSSR-1461	GTCCTCTCCTACGCCTCCTC	60.3			
	ATGCGATCCGAGTTCAGAAG				
mEgCIR2332	GAAGAAGAGCAAAAGAGAAG	55.0	*E. guineensis.*	*B. flabellifer*	[44, 45]
	GCTAGGTGAAAAATAAAGTT				
mEgCIR3295	TGCCTCCAGACAATCAC	55.0			
	GTAAGGCTTAACCAGATAAC				
mEgCIR3311	AATCCAAGTGGCCTACAG	55.0			
	CATGGCTTTGCTCAGTCA				
mEgCIR3413	AAAGCTATGGGGTGAAAGAT	55.0			
	TGGATAAGGGCGAGAAGAGA				
mEgCIR3477	CCTTCAAGCAAAGATACC	55.0			
	GGCACCAAACACAGTAA				
mEgCIR3592	GAGCCAAAACAGACTTCAA	55.0			
	ACCGTATATGACCCCTCTC				
mEgCIR3755	GCTCACCAAAAAGTGTTAAGTC	55.0			
	AGTTTCAACGGCAGGTATAT				
mEgCIR3788	TTGTATGACCAAAGACAGC	55.0			
	AGCGCAACATCAGACTA				
ESSR75	AGATGGTTGGAGATTTCATGGT	60.0	*E. guineensis*	*B. flabellifer*	[44, 45, 47]
	AACTTGAGGGTGCCATTACAAG				
ESSR76	CCATACCAGCAGAAGAGGATGT	60.0			
	CTGAAGGTCATAGGGGTCTCTG				
ESSR82R	CCCTCGACACCCATAGTTATTT	60.0			
	CTCGATTTCTGGCCTCTCATAC				
ESSR332	AGTTAATGTGTCAGGGCCAGTT	60.0			
	CTTGGTTCACTTGGGTGTGTC				
ESSR553	ATAAATTGTGCGAGGGGAAAAC	60.0			
	AGATCCGCGACAGGTCTTAAC				
ESSR566	GTGTCATCAAATTCGGTCCTTT	60.0			
	CGGTTCTTCTGCTGCTCTACTT				
ESSR609	AGGCGGTGATGAAGATGAAG	59.0			
	CTCCTCTCAAACAGAGTGGGAT				
ESSR650	GCCTTTTCTGGTTAATGGACTG	59.0			
	GTTTGTCTATGGATGATTGTGAGG				
ESSR652	CATACCGTCACCACTCAGAAAC	60.0			
	GCCGTCATTCTACCAGTTGAG				
ESSR673	TTCTGGCTACGAGCATAAGGA	59.0			
	TCAATAACCCTGGCTAAACACA				
ESSR681	TCTGAATTGTCGGAGTGGC	59.0			
	CATCCTTGCGTAAACAAAAGAG				
CNZ34	CATGTCGATAATTATACCCAA	55.0	*C. nucifera*	*B. flabellifer* *K.laciniosa* *Z. zalacca* *D.kurzianus* *C.simplicifolia* *C. mannan* *C. thwaitesii* *C. erectus* *C. palustris*	[46, 63]
	TGCAAATATGAATGCAAACAC				
CN2A5	AAGGTGAAATCTATGAACACA	53.2			
	GGCAGTAACACATTACACATG				
CNZ 12	TAGCTTCCTGAGATAAGATGC	54.6	*C. nucifera*	*B. flabellifer* *P. dactylifera* *E. guineensis*	[46, 64]
	GATCATGGAACGAAAACATTA				
CNZ 24	TCCTAAGCTCAATACTCACCA	55.0			
	CGCATTGATAAATACAAGCTT				
CNZ 18	ATGGTTCAGCCCTTAATAAAC	60.3			
	GAACTTTGAAGCTCCCATCAT				
CNZ 42	TGATACTCCTCTGTGATGCTT	55.5			
	GTAGATTGTGGGAGAGGAATG				
CN2A4	CAGGATGGTTCAAGCCCTTAA	61.0			
	GGTGGAAGAGGGAGAGATTGA				
CAC 21	AATTGTGTGACACGTAGCC	54.1	*C. nucifera*	*B. flabellifer*	[65, 66]
	GCATAACTCTTTCATAAGGGA				
CAC 71	ATAGCTCAAGTTGTTGCTAGG	54.2			
	ATATTGTCATGATTGAGCCTC				
CAC 84	TTGGTTTTTGTATGGAACTCT	54.4			
	AAATGCTAACATCTCAACAGC				
CN1H2	TTGATAGGAGAGCTTCATAAC	53.2	*C. nucifera*	*B. flabellifer* *P. dactylifera*	[65]
	ATCTTCTTTAATGCTCGGAGT				
PdAG-SSR	TCTGATTTCGTTTACTTCTTAGGA	58.0	*P. dactylifera*		[44]
	TTCATATTCAGTTGTCGGGTGTA				
mPdCIR015	AGCTGGCTCCTCCCTTCTTA	59.1			
	GCTCGGTTGGACTTGTTCT				
mPdCIR063	CTTTTATGTGGTCTGAGAGA	52.5			
	TCTCTGATCTTGGGTTCTGT				
mPdIRD1	CTCGGAAGGGTATGGACAAA	59.6	*P. dactylifera*	*P. reclinata* *P. roebelenii* *P. rupicola* *P. theophrasti* *H. thebaica* *L. carinensis* *C. humilis*	[67]
	TTGCCTTCGACGTGGTAGTA				
mPdIRD3	CATTGATCCAACACCACCAC	60.3			
	GCCAAAACCAGCTCTGGTAAC				
mPdIRD4	TTGGTGGCCTTTCTCAGAGT	59.8			
	TGGGATCAAAGTAGGGTTGG				
mPdIRD5	CTATCAGGATGGGGGTGATG	60.2			
	ACCCATCTGCATAGCTCCAG				
mPdIRD7	TGCAATACGATGGCAGAGTC	60.2			
	CCTTGCAAGTTTTCCACACC				
mPdIRD8	CTATTGGGTCCCTTGGTGAG	59.7			
	TGACTGCTCGTCATCAGGTC				
mPdIRD10	ATGCGTTCATCTCCCTTGAG	59.7			
	GCTGCAAACATCATCCTCAC				
mPdIRD11	GAGTTGGAGGCAAAACCAGA	59.8			
	CCACAAAACCCTTGTCTTCC				
mPdIRD14	GAGGGGTTCACGTTTGTGTC	60.9			
	GCACCAAGCACAAGAGCAAT				
mPdIRD15	CCGAGTCTGGCGAAGTAAAC	60.0			
	CTCCCCTTCCTCATCCTCTC				
mPdIRD16	CTGTCCGATCGAATTCTGC	50.7			
	GGACATCTCTTTGCGGTCAT				
mPdIRD17	GTGGGAGAAACCCGAAGAAT	60.2			
	CTGCTGCCTCATCTGCATT				
mPdIRD20	TTGAATGGTCCCCTGTAGGT	59.5			
	GTCCCAGCATGATTGCAGTA				
mPdIRD22	GGCTGTATGGGAAAGACCTG	59.5			
	CCTGCTGCATATTCTTCGTG				
mPdIRD24	GCTCCTGCAGAACCTGAAAC	59.9			
	GGACATCACCGTCCAATTCT				
mPdIRD25	CACTGGAAATTCAGGGCCTA	59.9			
	CCCAATTTCTCAGCCAAGAC				
mPdIRD26	CCTCCAGTTCATGCTTCTCC	60.0			
	GAGCAGACCCGACAGACAAT				
mPdIRD28	GAAACGGTATCGGGATGATG	59.7			
	TTAACGACGCCGTTTCCT				
mPdIRD29	GGCTCCACCATCATTGACA	60.3			
	AACAGCATCGACTGCCTTCT				
mPdIRD30	GCAGATGGTTGAAAGCTCCT	59.8			
	CCCCATTAACAGGATCAACG				
mPdIRD31	GCAGGTGGACTGCAAAATCT	60.0			
	CTATTGGGGTGCTGATCCAT				
mPdIRD32	AAGAAGACATTCCGGCTGGT	59.9			
	GCGGGTGTGTGATATTGATG				
mPdIRD33	GGAGCATACAGTGGGTTTGC	60.1			
	CAGCCTGGGAATGAGGATAG				
mPdIRD35	CAGCCCCTTACTCAGACTGG	59.6			
	CCCATAAGCTGATTGTGCTG				
mPdIRD36	GACACGTTGACGATGTGGAA	60.7			
	CCATTGCTGTTGAGGAGGAG				
mPdIRD37	TTTCCTGCTCGAAAGACACC	60.2			
	CTTAGCCAGCCTCCACACTC				
mPdIRD40	GAGAGATGCGTCAGGGAATC	59.2			
	CCAGAATCTTCCAAGCAAGC				
mPdIRD42	GAGGCAAAACTATGGGAAGC	59.5			
	TTCACTGGAGCAAGGGTAGG				
mPdIRD43	GCAGCCATTGCTTACAGTGA	60.2			
	TAAACTGCTGCCTTCCTTGG				
mPdIRD44	CAGATCCGGGAGATGATGAA	60.4			
	AGCAGGAGCAGCTGCATAA				
mPdIRD45	TAGCCTGTGCATGTTCGTTG	60.4			
	AACAGCAGCTGATGGTGATG				
mPdIRD46	ATGGGTCCATTGGAGGAACT	60.2			
	GACGGAGACCTTGACTGCTC				
mPcCIR10	ACCCCGGACGTGAGGTG	62.8	*P. dactylifera*		Cherif, Castillo and Aberlenc-Bertossi, unpublished data.
	CGTCGATCTCCTCCTTTGTCTC				
mPcCIR20	GCACGAGAAGGCTTATAGT	51.7			
	CCCCTCATTAGGATTCTAC				
mPcCIR32	CAAATCTTTGCCGTGAG	53.3			
	GGTGTGGAGTAATCATGTAGTAG				
mPcCIR35	ACAAACGGCGATGGGATTAC	60.8			
	CCGCAGCTCACCTCTTCTAT				
mPcCIR50	CTGCCATTTCTTCTGAC	50.6			
	CACCATGCACAAAAATG				
mPcCIR57	AAGCAGCAGCCCTTCCGTAG	62.0			
	GTTCTCACTCGCCCAAAAATAC				
mPcCIR85	GAGAGAGGGTGGTGTTATT	51.8			
	TTCATCCAGAACCACAGTA				
mPdIRD41	ATCTTCCATGCAGCCTCAAG	60.3			
	CAGGTCGTCCCGTCTCTAAA				
mPdIRD47	GTTGGCATCACTTCAGAGCA	60.1			
	GCTCTTTCGGTGCTAGTTGC				

For each marker, forward (top) and reverse primers (bottom) are provided

T_a_: average annealing temperature for each primer pair

Species names are abbreviated as follows: *P. roebelinii: Phoenix roebelinii; P. canariensis: Phoenix canariensis; Phoenix reclinata; H. thebaica*: *Hyphaene thebaica*; *L. carinensis: Livistona carinensis; C. humilis: Chamaerops humilis; K. laciniosa: Korthalsia laciniosa; Z. zalacca: Zalacca zalacca; D. kurzianus: Daemonorops kurzianus;C. simplicifolia: Calamus simplicifolia;C. mannan: Calamus mannan; C. thwaitesii: Calamus thwaitesii; C. erectus: Calamus erectus; C. palustris: Calamus palustris; P. rupicola: Phoenix rupicola; P. theophrasti: Phoenix theophrasti*

In any case, from the low number of successfully transferred microsatellite markers we could only identify one displaying polymorphism in our *B. aethiopum* test panel, making it impossible to rely on for analysis of genetic diversity. Still, the fact that so little microsatellite polymorphism (2 out of 18 amplifying primer pairs: 11.1%) could be detected in this subset of 20 palms sampled across different locations throughout Benin is somewhat surprising and its reasons remain to be elucidated. In addition to possibly being a symptom of habitat fragmentation, this low diversity might also result from the extremely long juvenile phase that has been attributed to this palm species. Indeed, floral maturity has been reported to occur 30 to 50 years after germination [68]. The manner of seed and pollen dispersal, which have so far not been studied extensively in *B. aethiopum*, might also play a role. Indeed, in pollen-mediated gene flow species, the distance the pollen travel is of importance in the occurrence of crossing between populations [69, 70].

Regarding the development of novel SSR markers, our results are similar to other studies based on the use of high-throughput sequencing techniques in species where very little information is available [22, 71]. We identified 57 microsatellite loci, from which we selected 11 markers displaying polymorphism that were used to assess the genetic structure of *B. aethiopum* sampled from different sites in Benin. We find low genetic diversity, with an average He value (0.354) that is substantially below those reported for *B. flabellifer* (0.417) [45] and for other non-timber forest products such as *Khaya senegalensis* (0.53) [72] and *Phyllanthus sp.* (0.607 and 0.582 for *Phyllanthus emblica* and *Phyllanthus indofischeri* respectively [73]. The positive F value that we observed in the majority (6 out of 9) of locations in the present study indicates an overall deficiency of heterozygotes across sites. This deviation from the Hardy-Weinberg equilibrium (HWE) might reflect low gene flow through pollen and seed dissemination, leading to crosses between related individuals, as supported by the low average number of migrants between sites. Accordingly, our data reveal limited genetic distances among collection sites, with values that are lower than those reported for others palm species. Indeed for *B. flabellifer*, genetic distances ranged from 0.716 to 0.957 [74] and among natural *E. guineensis* accessions an average of 0.769 was observed [75]. Both our Fst values and AMOVA analysis point to intra-site differentiation as being the main source of genetic variation.

As illustrated by the global agreement between our PCoA and Bayesian analyses, Beninese *B. aethiopum* samples cluster into two main groups that are mostly dependent on geo-climatic regions and geographic distances between collection sites, although the correlation between genetic and geographic distance is poorly significant. There might be further genetic separation between Southern *B. aethiopum* samples and those from the Central sites of Agoua and Biguina, resulting in the splitting of one group into two subgroups. However, with our current dataset it is not possible to achieve this level of discrimination in our analyses. Additional sampling campaigns from intermediate locations in the Central and Northern regions will be necessary in order to make progress on the subject.

Among the nine locations studied in Benin, samples from Savè appear to be the most diversified (He = 0.451) and constitute the exception to the general distribution according to geographical distances. This site located in the Sudano-Guinean transition zone of Benin is currently the most active for the production of *B. aethiopum* hypocotyls, and it acts as a supplier for the whole national territory ([76]; V.K. Salako, personal communication), suggesting that it might be the largest population of *B. aethiopum* in the country. Moreover, individuals sampled in Savè appear to be genetically distinct from those sampled in other locations of the Central region and closer to those originating from the Northern region, despite the considerable geographical distances involved in the latter case. A part of the explanation for the genetic distance observed between the Western (i.e. Biguina and Agoua) and the Eastern (Savè) collection sites within the Central region may reside in their physical separation by the Ouémé river, which further forms a natural corridor between Savè and the sites of Trois Rivières and Malanville in the North-East (see Fig. 1) [77]. We postulate that seed dispersal by humans and/or animals along this corridor might have played a major role in the observed pattern of genetic diversity and explain the singularity observed in Savè. As a matter of fact, members of the Bariba ethnic group, who live in the Eastern part of the country up to Malanville, share strong historical ties with the Shabè people from Savè, and exchanges between both groups are frequent [78]. The same corridor is also used annually for transhumance by the Fulani people [79], for whom *B. aethiopum* is an important plant: the role of their mobility in the dispersal of the plant, similar to what has been proposed for *Caesalpina bonduc* [80], is therefore plausible. Regarding the impact of animal migrations, Salako et al. [31, 32] detected the presence of *B. aethiopum* seeds in elephant dungs and hypothesized that elephants may have played important role in the seed dissemination for this species through fruit consumption and long-distance herd migrations. In support to this assumption, Savè is part of a continuous forest corridor connecting with the Northern region that was likely used by elephants in their migrations. Up until 1982, the seasonal occurrence of the animal has been reported in the Wari-Maro forest of Central Benin [81].

The specific microsatellite markers developed in this study from the partial genomic sequencing of *B. aethiopum* appear to be efficient to assess the genetic diversity and population structure of this species. Additionally, and provided that genome divergence is not too extensive to allow marker transferability, our SSR markers may also been used in a palm species that belongs to the same genus and that is reported to share parts of its distribution area, namely *Borassus akeassii* B.O.G., which has long been confused with *B. aethiopum* due to its similar morphology [82]. High-throughput sequencing techniques are an effective way of developing new microsatellite markers in plant species without significant molecular data. The increasing technical performances and financial affordability of these technologies make it feasible to overcome the difficulties arising in case studies such as ours, where marker transfer was proved to be limited or ineffective.

## Conclusions

To our knowledge, the data presented in the present article constitute the first sizeable molecular resource available for *Borassus aethiopum*, which we have made available to the scientific community at large in order to facilitate the implementation of an increasing number of studies on this palm species. Using 11 newly identified SSR markers, we have also performed the first analysis of the genetic diversity of *B. aethiopum* in an African country, which we see as a first step towards the elaboration of an evidence-based strategy for sustainable resource management and preservation in Benin. Our results support the hypothesis that pollen and seed dispersal mainly occur within sites, leading to crosses among related individuals. The exception to this general rule in the region of Savè (Center) seems to indicate long-range transfer of genes as a result to animal and/or human movements towards and from forest reserves of the North. Further research into the characteristics of these migrations and their impact on gene flow among *B. aethiopum* populations is required in order to confirm this assumption. As a complement to the present work, the acquisition of agro-morphological data is currently under way, in a bid to elucidate the reproductive development and breeding system of the species. As a longer-term perspective, we also plan to extend our analysis of *B. aethiopum* diversity to the West African sub-region, and leverage the data acquired to improve knowledge of other species within the *Borassus* genus, and of palms diversity as a whole.

## Methods

### Plant material sampling and DNA extraction

Samples of *Borassus aethiopum* were collected from wild populations in nine distinct sites (three located in protected forest areas, six in farmlands) that were distant from each other by at least 50 km and which spanned the three main climatic regions encountered in Benin (Fig. 1). According to White [83], Benin covers three contrasted climatic regions which are the Sudanian region in the North, the Sudano-Guinean region in the Center and the Guineo-Congolian region in the South. Along a South-North gradient, the rainfall regime switches from bimodal to unimodal, the climate becomes globally drier [29] and the density of *B. aethiopum* distribution increases [31]. At each location, young leaves from 10 male and 10 female adult trees separated by at least 100 m were collected and stored in plastic bags containing silica gel until further processing. The complete list of samples and their characteristics is available in Additional file 2.

Genomic DNA was extracted from 250 mg of leaves ground to powder under liquid nitrogen using the Chemagic DNA Plant Kit (Perkin Elmer, Germany), according to the manufacturer’s instructions on a KingFisher Flex™ (Thermo Fisher Scientific, USA) automated DNA purification workstation. Final DNA concentration was assessed fluorometrically with the GENios Plus reader (TECAN) using bis-benzimide H 33258 (Sigma-Aldrich) as a fluorochrome.

#### Transferability of palms microsatellite markers: selection and amplification

A total of 80 SSR markers from previous studies were selected for assessment of their transferability to *B. aethiopum*: 44 developed for *Phoenix dactylifera* [67]; 25 developed for *Elaeis guineensis* [44, 62]; and 11 developed for *Cocos nucifera* [65]. The respective sequences and origins of these primer sets are displayed in Table 8.

Transferability of the 80 palm SSR markers was assessed on a representative subset of 20 *B. aethiopum* individuals sampled at the different locations, plus four positive controls from each.

source species for these markers (i.e. *P. dactylifera, C. nucifera,* and *E. guineensis*). Microsatellite amplification was performed with a modification of the M13-tailed Primers protocol [63] adapted to the use of fluorescent labelling [64]. The PCR reaction was performed on 20 ng of leaf DNA in volume of 20 μL with the following final concentrations or amounts: 1X PCR buffer, 200 μM dNTP, 2 mM MgCl_2_, 0.4 pmol M13-tailed forward primer, 4 pmol M13 primer, (5′-CACGACGTTGTAAAACGAC-3′) fluorescently labeled at the 5′ end with FAM, HEX or TAMR, 4 pmol reverse primer, and 0.5 U of KAPA *Taq* polymerase (Sigma-Aldrich). The following program was used: 3 min of initial denaturation at 95 °C, followed by 35 cycles of 30 s at 95 °C, 30 s at 50 °C and 72 °C for 1 min and a final extension at 72 °C for 5 min. The resulting amplification products were then diluted to 1/10th, mixed with 0.5 μL of an internal size standard (GeneScan 500 ROX, Thermo Fisher Scientific), and denatured for 5 min at 94 °C prior to separation through capillary electrophoresis on an Applied Biosystems 3500 Genetic Analyzer (Thermo Fisher Scientific). Amplification products visualization was performed using the GeneMapper software version 3.7 (Applied Biosystems).

#### De novo identification of microsatellite loci in the *B. aethiopum* genome, marker selection and diversity analysis

One *B. aethiopum* leaf sample (originating from the Togbin site) was randomly selected and used for genomic DNA purification according to the protocol of Mariac et al. [84]. The DNA was then used for the construction of an Illumina paired-end library, as described in Mariac et al. [85], before high-throughput sequencing on a MiSeq v3 platform (Illumina; average read size 250 bp). Demultiplexing of the raw data output was performed using the Maillol script (https://github.com/maillol/demultadapt), with a 0-mistmatch threshold. Adapters were eliminated using Cutadapt version 1.10 [86]. (http://code.google.com/p/cutadapt/) with the following parameters: overlap length = 7, minimum length = 35 and quality = 20. High-quality reads (Q > 30) were filtered using the following script: https://github.com/SouthGreenPlatform/arcad-hts/blob/master/scripts/arcad_hts_2_Filter_Fastq_On_Mean_Quality.pl and the resulting filtered reads were deposited into GenBank under BioProject ID PRJNA576413. Paired-end reads were then merged using FLASH version 1.2.11 (https://github.com/SouthGreenPlatform/arcad-hts/blob/master/scripts/arcad_hts_3_synchronized_paired_fastq.pl). Finally, microsatellite motif detection and specific primer design were carried out after elimination of redundant sequences using the QDD software version 3.1.2 [87] with default settings (detailed in Additional file 3).

Using selected primer pairs, test amplifications were performed with two randomly selected *B. aethiopum* DNA samples, then primers showing successful amplification were further tested for polymorphism detection among seven randomly selected DNA samples. The M13 Tailed Primers protocol described previously was used, with the following program: 3 min of initial denaturation at 95 °C, followed by 35 cycles of 30 s at 95 °C, 30 s at 55 °C and 72 °C for 1 min and a final extension at 72 °C for 5 min. Separation and vizualization of amplification products were performed as described previously. Finally, the primer pairs enabling successful and unambiguous amplification of polymorphic bands were used for the analysis of genetic diversity among the complete set of 180 *B. aethiopum* individuals under the same PCR conditions.

### Data analysis

Amplification products were scored using the GeneMapper software version 3.7 (Applied Biosystems) and only unambiguous amplification products were considered for data analysis. Genetic diversity parameters were calculated for each locus and each sampling location using the GenAlEx software version 6.502 [88]. Expected heterozygosity (He) was calculated using the formula:

$$ \mathrm{He}=1-\sum {\mathrm{p}}_{\dot{\mathrm{i}}}^2 $$

where p_i_ is the frequency of each allele. The fixation index (F) was calculated as:

$$ \mathrm{F}=1-\frac{{\mathrm{H}}_0}{\mathrm{H}\mathrm{e}} $$

where Ho is observed heterozygosity and He is expected heterozygosity [89].

F-statistics analysis assessing genetic differentiation (Fst), genetic identity, number of migrants (Nm) [90] and non-hierarchical analysis of molecular variance (AMOVA) for estimating genetic differentiation within and among locations were performed with the same software. Allelic richness was calculated using the SPAGeDi software version 1.5 (http://ebe.ulb.ac.be/ebe/SPAGeDi.html [91];). Consecutively to K determination (see below), successive hierarchical AMOVA analyses were carried out with K = 2 and K = 3. The Mantel permutation test was used for assessing the correlation between genetic and geographic distances between sampling sites [92, 93]. Two Principal Coordinates Analyses (PCoA) enabling the visualization of genetic variation distribution across individuals and sampling sites, respectively, were performed using GenAlEx.

The STRUCTURE software version 2.3.4 [94] was used for the determination of the most probable number of clusters for population structure (K value). Using the admixture model, eight simulations were performed for each inferred K value, with a running length composed of 300,000 burn-in periods and 50,000 Markov chain Monte Carlo (MCMC) replicates. The output from this analysis was then used as input in the Structure HARVESTER online program version 0.6.94 (http://taylor0.biology.ucla.edu/structureHarvester/) to determine the optimal value of K using the ΔK method of Evanno et al. [95] and allowing for different estimates of K in accordance with Janes et al [50]. Based on the resulting values of K, a clustering analysis of the studied sampling sites was performed and graphical output was generated using CLUMPAK’s main pipeline (http://clumpak.tau.ac.il [96];). In order to further assess genetic clustering, a UPGMA tree based on Fst values using 1000 bootstrap replications was constructed using the POPTREE2 software [97].

## Supplementary Information

**Additional file 1. **List and characteristics of putative microsatellite loci identified in the genome of *Borassus aethiopum* through QDD analysis. Characteristics (basic motif, length) of the microsatellite loci and of the primer pairs (length, position, Tm, stability, amplicon size and sequence) designed for their targeted amplification.

**Additional file 2. **List of sampled *Borassus aethiopum* individuals. M, F: male or female palm, respectively. All geographic coordinates are provided as North from the Equator (latitude) and East from the Greenwich meridian (longitude), respectively.

**Additional file 3.** Default QDD software settings. Parameters used in the QDD detection of microsatellite loci.

**Additional file 4.** Results of the Bayesian cluster analysis with variable values of K. Graphical summary generated from STRUCTURE results by CLUMPAK’s main pipeline with values of K ranging from 1 to 10.

## Data Availability

Data generated from genome sequencing (filtered reads) were deposited into GenBank under BioProject ID PRJNA576413. Capillary electrophoresis profiles are available upon reasonable request to the Corresponding Author. All other data generated or analyzed during this study are included in this published article (and its supplementary information files).

## Abbreviations

AMOVA: Analysis of molecular variance
F: Fixation index
F_st_: Inter-population genetic differentiation coefficient
He: Expected Heterozygosity
Ho: Observed Heterozygosity
HWE: Hardy-Weinberg equilibrium
Na: Average number of different alleles
Ne: Effective number of alleles
N_m_: Number of migrants
PCoA: Principal coordinate analysis
SSR: Simple sequence repeat
UPGMA: Unweighted pair-group method with arithmetic mean
