# Supplementary material for: Transferability, development of simple sequence repeat (SSR) markers and application to the analysis of genetic diversity and population structure of the African fan palm (Borassus aethiopum Mart.) in Benin
Source: BMC Genet. 2020 Dec 3;21:145. doi: 10.1186/s12863-020-00955-y (PMC7713368; doi:10.1186/s12863-020-00955-y)
Supplement: Supplementary file 2 — Additional file 2. List of sampled Borassus aethiopum individuals. M, F: male or female palm, respectively. All geographic coordinates are provided as North from the Equator (latitude) and East from the Greenwich meridian (longitude), respectively. [file 12863_2020_955_MOESM2_ESM.docx]

**S1 Table:** List of sampled *B. aethiopum* individuals.

| **N°** | **Collection site ID** | **Sample ID** | **Site** | **Sex** | **Latitude** | **Longitude** |
| --- | --- | --- | --- | --- | --- | --- |
| 1 | COLL1 | Bat1 | TOGBIN | M | 06°20'55.9'' | 002°19'15.5'' |
| 2 | COLL1 | Bat2 | TOGBIN | M | 06°20'55.9'' | 002°19'15.1'' |
| 3 | COLL1 | Bat3 | TOGBIN | M | 06°21'00.8'' | 002°19'09.8'' |
| 4 | COLL1 | Bat4 | TOGBIN | M | 06°21'09.1'' | 002°19'06.1'' |
| 5 | COLL1 | Bat5 | TOGBIN | F | 06°20'52.4'' | 002°18'47.0'' |
| 6 | COLL1 | Bat6 | TOGBIN | M | 06°20'56.8'' | 002°18'43.7'' |
| 7 | COLL1 | Bat7 | TOGBIN | F | 06°20'54.0'' | 002°18'37.8'' |
| 8 | COLL1 | Bat8 | TOGBIN | F | 06°20'53.6'' | 002°18'37.6'' |
| 9 | COLL1 | Bat9 | TOGBIN | M | 06°20'55.1'' | 002°18'35.8'' |
| 10 | COLL1 | Bat10 | TOGBIN | F | 06°20'54.7'' | 002°18'34.7'' |
| 11 | COLL1 | Bat11 | TOGBIN | M | 06°20'57.9'' | 002°18'34.5'' |
| 12 | COLL1 | Bat12 | TOGBIN | F | 06°20'58.9'' | 002°18'36.0'' |
| 13 | COLL1 | Bat13 | TOGBIN | F | 06°21'02.5'' | 002°18'36.4'' |
| 14 | COLL1 | Bat14 | TOGBIN | M | 06°21'01.9'' | 002°18'35.8'' |
| 15 | COLL1 | Bat15 | TOGBIN | F | 06°21'00.4'' | 002°18'33.3'' |
| 16 | COLL1 | Bat16 | TOGBIN | M | 06°21'01.8'' | 002°18'32.6'' |
| 17 | COLL1 | Bat17 | TOGBIN | F | 06°21'00.3'' | 002°18'30.7'' |
| 18 | COLL1 | Bat18 | TOGBIN | M | 06°20'58.1'' | 002°18'27.1'' |
| 19 | COLL1 | Bat19 | TOGBIN | F | 06°20'57.9'' | 002°18'19.9'' |
| 20 | COLL1 | Bat20 | TOGBIN | F | 06°20'57.6'' | 002°18'20.7'' |
| 21 | COLL2 | Bat21 | HOUNVIATOUIN | M | 06°44'11.2'' | 001°59'01.5'' |
| 22 | COLL2 | Bat22 | HOUNVIATOUIN | M | 06°44'11.4'' | 001°59'01.7'' |
| 23 | COLL2 | Bat23 | HOUNVIATOUIN | M | 06°44'12.0'' | 001°59'01.8'' |
| 24 | COLL2 | Bat24 | HOUNVIATOUIN | M | 06°44'11.8'' | 001°59'03.6'' |
| 25 | COLL2 | Bat25 | HOUNVIATOUIN | M | 06°44'12.5'' | 001°59'04.9'' |
| 26 | COLL2 | Bat26 | HOUNVIATOUIN | M | 06°44'14.8'' | 001°59'04.3'' |
| 27 | COLL2 | Bat27 | HOUNVIATOUIN | F | 06°44'17.0'' | 001°59'02.6'' |
| 28 | COLL2 | Bat28 | HOUNVIATOUIN | M | 06°44'17.0'' | 001°59'00.7'' |
| 29 | COLL2 | Bat29 | HOUNVIATOUIN | M | 06°43'50.3'' | 001°59'06.4'' |
| 30 | COLL2 | Bat30 | HOUNVIATOUIN | M | 06°43'51.3'' | 001°59'03.8'' |
| 31 | COLL2 | Bat31 | HOUNVIATOUIN | M | 06°43'47.4'' | 001°59'06.8'' |
| 32 | COLL2 | Bat32 | HOUNVIATOUIN | F | 06°43'48.0'' | 001°59'05.8'' |
| 33 | COLL2 | Bat33 | HOUNVIATOUIN | F | 06°43'46.5'' | 001°59'07.6'' |
| 34 | COLL2 | Bat34 | HOUNVIATOUIN | F | 06°43'46.2'' | 001°59'08.3'' |
| 35 | COLL2 | Bat35 | HOUNVIATOUIN | F | 06°43'43.7'' | 001°59'09.7'' |
| 36 | COLL2 | Bat36 | HOUNVIATOUIN | F | 06°43'43.4'' | 001°59'06.9'' |
| 37 | COLL2 | Bat37 | HOUNVIATOUIN | F | 06°43'44.1'' | 001°59'06.1'' |
| 38 | COLL2 | Bat38 | HOUNVIATOUIN | F | 06°43'53.9'' | 001°59'03.4'' |
| 39 | COLL2 | Bat39 | HOUNVIATOUIN | F | 06°43'52.8'' | 001°59'00.4'' |
| 40 | COLL2 | Bat40 | HOUNVIATOUIN | F | 06°43'54.9'' | 001°58'59.9'' |
| 41 | COLL3 | Bat41 | SAVE | F | 08°04'12.2'' | 002°31'07.6'' |
| 42 | COLL3 | Bat42 | SAVE | M | 08°04'12.2'' | 002°31'07.7'' |
| 43 | COLL3 | Bat43 | SAVE | F | 08°05'30.8'' | 002°32'10.1'' |
| 44 | COLL3 | Bat44 | SAVE | M | 08°05'30.2'' | 002°32'17.1'' |
| 45 | COLL3 | Bat45 | SAVE | M | 08°05'32.5'' | 002°32'15.8'' |
| 46 | COLL3 | Bat46 | SAVE | F | 08°05'33.0'' | 002°32'10.7'' |
| 47 | COLL3 | Bat47 | SAVE | M | 08°06'00.5'' | 002°32'39.3'' |
| 48 | COLL3 | Bat48 | SAVE | F | 08°05'58.0'' | 002°32'43.2'' |
| 49 | COLL3 | Bat49 | SAVE | F | 08°05'58.4'' | 002°32'38.7'' |
| 50 | COLL3 | Bat50 | SAVE | F | 08°06'18.5'' | 002°32'43.6'' |
| 51 | COLL3 | Bat51 | SAVE | M | 08°07'38.6'' | 002°34'45.9'' |
| 52 | COLL3 | Bat52 | SAVE | M | 08°07'39.9'' | 002°34'48.6'' |
| 53 | COLL3 | Bat53 | SAVE | M | 08°07'17.6'' | 002°37'03.5'' |
| 54 | COLL3 | Bat54 | SAVE | M | 08°07'18.6'' | 002°37'05.4'' |
| 55 | COLL3 | Bat55 | SAVE | F | 08°01'38.3'' | 002°28'35.9'' |
| 56 | COLL3 | Bat56 | SAVE | M | 08°10'25.9'' | 002°37'16.0'' |
| 57 | COLL3 | Bat57 | SAVE | M | 08°08'19.6'' | 002°35'54.1'' |
| 58 | COLL3 | Bat58 | SAVE | F | 08°08'11.9'' | 002°36'18.7'' |
| 59 | COLL3 | Bat59 | SAVE | F | 08°08'09.8'' | 002°36'17.7'' |
| 60 | COLL3 | Bat60 | SAVE | F | 08°07'47.2'' | 002°34'07.5'' |
| 61 | COLL4 | Bat61 | BIGUINA | F | 08°44'46.4'' | 001°38'10.3'' |
| 62 | COLL4 | Bat62 | BIGUINA | M | 08°44'48.7'' | 001°38'05.6'' |
| 63 | COLL4 | Bat63 | BIGUINA | F | 08°44'48.9'' | 001°38'05.3'' |
| 64 | COLL4 | Bat64 | BIGUINA | M | 08°44'49.3'' | 001°38'04.8'' |
| 65 | COLL4 | Bat65 | BIGUINA | F | 08°44'51.8'' | 001°38'05.1'' |
| 66 | COLL4 | Bat66 | BIGUINA | M | 08°44'52.5'' | 001°38'03.8'' |
| 67 | COLL4 | Bat67 | BIGUINA | F | 08°44'52.5'' | 001°38'03.3'' |
| 68 | COLL4 | Bat68 | BIGUINA | M | 08°44'53.1'' | 001°38'02.9'' |
| 69 | COLL4 | Bat69 | BIGUINA | F | 08°44'53.2'' | 001°38'03.0'' |
| 70 | COLL4 | Bat70 | BIGUINA | M | 08°44'53.4'' | 001°38'02.5'' |
| 71 | COLL4 | Bat71 | BIGUINA | F | 08°44'58.8'' | 001°38'04.4'' |
| 72 | COLL4 | Bat72 | BIGUINA | M | 08°44'59.7'' | 001°38'03.4'' |
| 73 | COLL4 | Bat73 | BIGUINA | M | 08°45'01.0'' | 001°38'04.3'' |
| 74 | COLL4 | Bat74 | BIGUINA | F | 08°45'03.2'' | 001°38'04.1'' |
| 75 | COLL4 | Bat75 | BIGUINA | F | 08°45'03.8'' | 001°38'04.6'' |
| 76 | COLL4 | Bat76 | BIGUINA | M | 08°45'04.5'' | 001°38'04.8'' |
| 77 | COLL4 | Bat77 | BIGUINA | M | 08°45'05.0'' | 001°38'05.0'' |
| 78 | COLL4 | Bat78 | BIGUINA | F | 08°45'05.5'' | 001°38'05.0'' |
| 79 | COLL4 | Bat79 | BIGUINA | M | 08°45'05.1'' | 001°38'03.8'' |
| 80 | COLL4 | Bat80 | BIGUINA | F | 08°45'05.3'' | 001°38'06.6'' |
| 81 | COLL5 | Bat81 | PENDJARI | M | 11°28'03.6'' | 001°33'58.6'' |
| 82 | COLL5 | Bat82 | PENDJARI | M | 11°28'03.6'' | 001°33'59.2'' |
| 83 | COLL5 | Bat83 | PENDJARI | M | 11°28'04.2'' | 001°33'59.3'' |
| 84 | COLL5 | Bat84 | PENDJARI | F | 11°28'04.6'' | 001°33'59.4'' |
| 85 | COLL5 | Bat85 | PENDJARI | F | 11°28'04.3'' | 001°33'59.9'' |
| 86 | COLL5 | Bat86 | PENDJARI | F | 11°28'03.5'' | 001°34'00.6'' |
| 87 | COLL5 | Bat87 | PENDJARI | F | 11°28'03.4'' | 001°34'00.9'' |
| 88 | COLL5 | Bat88 | PENDJARI | F | 11°28'03.6'' | 001°34'01.0'' |
| 89 | COLL5 | Bat89 | PENDJARI | M | 11°28'03.0'' | 001°33'59.8'' |
| 90 | COLL5 | Bat90 | PENDJARI | M | 11°28'01.9'' | 001°33'59.5'' |
| 91 | COLL5 | Bat91 | PENDJARI | M | 11°28'00.9'' | 001°33'57.5'' |
| 92 | COLL5 | Bat92 | PENDJARI | M | 11°28'01.6'' | 001°33'56.0'' |
| 93 | COLL5 | Bat93 | PENDJARI | M | 11°28'01.7'' | 001°33'55.0'' |
| 94 | COLL5 | Bat94 | PENDJARI | M | 11°27'58.3'' | 001°33'54.4'' |
| 95 | COLL5 | Bat95 | PENDJARI | F | 11°27'57.6'' | 001°33'56.5'' |
| 96 | COLL5 | Bat96 | PENDJARI | F | 11°27'58.4'' | 001°33'55.8'' |
| 97 | COLL5 | Bat97 | PENDJARI | F | 11°27'58.7'' | 001°33'55.9'' |
| 98 | COLL5 | Bat98 | PENDJARI | F | 11°27'39.0'' | 001°34'01.2'' |
| 99 | COLL5 | Bat99 | PENDJARI | F | 11°27'38.7'' | 001°34'01.0'' |
| 100 | COLL5 | Bat100 | PENDJARI | M | 11°27'39.2'' | 001°33'56.9'' |
| 101 | COLL6 | Bat101 | PINGOU | M | 10°41'39.2'' | 001°06'52.9'' |
| 102 | COLL6 | Bat102 | PINGOU | F | 10°41'40.9'' | 001°06'53.2'' |
| 103 | COLL6 | Bat103 | PINGOU | F | 10°41'41.2'' | 001°06'53.4'' |
| 104 | COLL6 | Bat104 | PINGOU | M | 10°41'44.9'' | 001°06'47.6'' |
| 105 | COLL6 | Bat105 | PINGOU | F | 10°41'44.6'' | 001°06'46.5'' |
| 106 | COLL6 | Bat106 | PINGOU | F | 10°41'44.3'' | 001°06'46.0'' |
| 107 | COLL6 | Bat107 | PINGOU | F | 10°41'45.5'' | 001°06'46.2'' |
| 108 | COLL6 | Bat108 | PINGOU | F | 10°41'44.4'' | 001°06'44.3'' |
| 109 | COLL6 | Bat109 | PINGOU | M | 10°41'44.3'' | 001°06'44.1'' |
| 110 | COLL6 | Bat110 | PINGOU | F | 10°41'44.9'' | 001°06'43.9'' |
| 111 | COLL6 | Bat111 | PINGOU | M | 10°41'45.8'' | 001°06'43.0'' |
| 112 | COLL6 | Bat112 | PINGOU | F | 10°41'45.6'' | 001°06'43.5'' |
| 113 | COLL6 | Bat113 | PINGOU | F | 10°41'46.5'' | 001°06'42.7'' |
| 114 | COLL6 | Bat114 | PINGOU | F | 10°41'46.8'' | 001°06'43.0'' |
| 115 | COLL6 | Bat115 | PINGOU | M | 10°41'47.8'' | 001°06'41.6'' |
| 116 | COLL6 | Bat116 | PINGOU | M | 10°41'47.4'' | 001°06'40.0'' |
| 117 | COLL6 | Bat117 | PINGOU | M | 10°41'48.0'' | 001°06'39.5'' |
| 118 | COLL6 | Bat118 | PINGOU | M | 10°41'48.5'' | 001°06'40.1'' |
| 119 | COLL6 | Bat119 | PINGOU | M | 10°41'50.4'' | 001°06'41.5'' |
| 120 | COLL6 | Bat120 | PINGOU | M | 10°41'49.1'' | 001°06'.42.2'' |
| 121 | COLL7 | Bat121 | MALANVILLE | M | 11°49'10.8'' | 003°23'54.0'' |
| 122 | COLL7 | Bat122 | MALANVILLE | M | 11°49'11.4'' | 003°23'53.3'' |
| 123 | COLL7 | Bat123 | MALANVILLE | M | 11°49'11.6'' | 003°23'53.0'' |
| 124 | COLL7 | Bat124 | MALANVILLE | F | 11°49'10.8'' | 003°23'53.6'' |
| 125 | COLL7 | Bat125 | MALANVILLE | M | 11°49'09.7'' | 003°23'55.6'' |
| 126 | COLL7 | Bat126 | MALANVILLE | M | 11°49'09.5'' | 003°23'55.6'' |
| 127 | COLL7 | Bat127 | MALANVILLE | M | 11°49'09.7'' | 003°23'55.7'' |
| 128 | COLL7 | Bat128 | MALANVILLE | M | 11°49'10.1'' | 003°23'55.4'' |
| 129 | COLL7 | Bat129 | MALANVILLE | M | 11°49'09.9'' | 003°23'55.3'' |
| 130 | COLL7 | Bat130 | MALANVILLE | F | 11°49'10.0'' | 003°23'55.6'' |
| 131 | COLL7 | Bat131 | MALANVILLE | F | 11°49'10.3'' | 003°23'55.4'' |
| 132 | COLL7 | Bat132 | MALANVILLE | M | 11°49'10.1'' | 003°23'55.7'' |
| 133 | COLL7 | Bat133 | MALANVILLE | F | 11°49'09.5'' | 003°23'55.4'' |
| 134 | COLL7 | Bat134 | MALANVILLE | F | 11°49'06.6'' | 003°23'53.3'' |
| 135 | COLL7 | Bat135 | MALANVILLE | M | 11°49'06.6'' | 003°23'53.1'' |
| 136 | COLL7 | Bat136 | MALANVILLE | F | 11°49'06.2'' | 003°23'53.7'' |
| 137 | COLL7 | Bat137 | MALANVILLE | F | 11°49'09.0 | 003°23'50.9'' |
| 138 | COLL7 | Bat138 | MALANVILLE | F | 11°49'09.5'' | 003°23'50.9'' |
| 139 | COLL7 | Bat139 | MALANVILLE | F | 11°49'09.9'' | 003°23'50.6'' |
| 140 | COLL7 | Bat140 | MALANVILLE | F | 11°49'10.2'' | 003°23'51.2'' |
| 141 | COLL8 | Bat141 | AGOUA | F | 08°17'48.2" | 001°43'38.3" |
| 142 | COLL8 | Bat142 | AGOUA | M | 08°17'47.7" | 001°43'38.5" |
| 143 | COLL8 | Bat143 | AGOUA | M | 08°17'46.8" | 001°43'40.0" |
| 144 | COLL8 | Bat144 | AGOUA | F | 08°17'39.9" | 001°43'42.8" |
| 145 | COLL8 | Bat145 | AGOUA | F | 08°17'40.3" | 001°43'43.4" |
| 146 | COLL8 | Bat146 | AGOUA | F | 08°17'42.6" | 001°43'40.5" |
| 147 | COLL8 | Bat147 | AGOUA | F | 08°17'42.6" | 001°43'40.2" |
| 148 | COLL8 | Bat148 | AGOUA | M | 08°17'42.3" | 001°43'40.8" |
| 149 | COLL8 | Bat149 | AGOUA | M | 08°17'41.2" | 001°43'42.2" |
| 150 | COLL8 | Bat150 | AGOUA | M | 08°17'41.2" | 001°43'42.0" |
| 151 | COLL8 | Bat151 | AGOUA | F | 08°17'48.2" | 001°43'40.4" |
| 152 | COLL8 | Bat152 | AGOUA | M | 08°17'40.7" | 001°43'40.6" |
| 153 | COLL8 | Bat153 | AGOUA | M | 08°17'42.4" | 001°43'40.4" |
| 154 | COLL8 | Bat154 | AGOUA | M | 08°17'43.1" | 001°43'41.4" |
| 155 | COLL8 | Bat155 | AGOUA | M | 08°17'46.3" | 001°43'39.0" |
| 156 | COLL8 | Bat156 | AGOUA | M | 08°17'46.9" | 001°43'38.5" |
| 157 | COLL8 | Bat157 | AGOUA | F | 08°17'53.4" | 001°43'33.5" |
| 158 | COLL8 | Bat158 | AGOUA | F | 08°17'54.1" | 001°43'33.6" |
| 159 | COLL8 | Bat159 | AGOUA | F | 08°17'54.2" | 001°43'34.6" |
| 160 | COLL8 | Bat160 | AGOUA | F | 08°17'57.8" | 001°43'38.6" |
| 161 | COLL9 | Bat161 | TROIS RIVIERES | F | 10°38'15.5" | 002°55'40.3" |
| 162 | COLL9 | Bat162 | TROIS RIVIERES | F | 10°38'20.7" | 002°55'38.4" |
| 163 | COLL9 | Bat163 | TROIS RIVIERES | F | 10°38'20.5" | 002°55'40.9" |
| 164 | COLL9 | Bat164 | TROIS RIVIERES | F | 10°38'33.5" | 002°55'45.5" |
| 165 | COLL9 | Bat165 | TROIS RIVIERES | F | 10°38'44.5" | 002°55'40.4" |
| 166 | COLL9 | Bat166 | TROIS RIVIERES | F | 10°38'40.7" | 002°55'39.6" |
| 167 | COLL9 | Bat167 | TROIS RIVIERES | F | 10°38'35.6" | 002°55'40.1" |
| 168 | COLL9 | Bat168 | TROIS RIVIERES | F | 10°38'34.7" | 002°55'37.5" |
| 169 | COLL9 | Bat169 | TROIS RIVIERES | F | 10°38'37.8" | 002°55'42.1" |
| 170 | COLL9 | Bat170 | TROIS RIVIERES | F | 10°38'47.0" | 002°55'45.8" |
| 171 | COLL9 | Bat171 | TROIS RIVIERES | M | 10°30'21.2" | 002°55'39.7" |
| 172 | COLL9 | Bat172 | TROIS RIVIERES | M | 10°38'20.7" | 002°55'40.5" |
| 173 | COLL9 | Bat173 | TROIS RIVIERES | M | 10°38'22.5" | 002°55'42.7" |
| 174 | COLL9 | Bat174 | TROIS RIVIERES | M | 10°38'23.8" | 002°55'37.8" |
| 175 | COLL9 | Bat175 | TROIS RIVIERES | M | 10°38'24.7" | 002°55'38.9" |
| 176 | COLL9 | Bat176 | TROIS RIVIERES | M | 10°38'25.0" | 002°55'41.7" |
| 177 | COLL9 | Bat177 | TROIS RIVIERES | M | 10°38'27.9" | 002°55'40.5" |
| 178 | COLL9 | Bat178 | TROIS RIVIERES | M | 10°38'32.4" | 002°55'35.8" |
| 179 | COLL9 | Bat179 | TROIS RIVIERES | M | 10°38'41.7" | 002°55'42.1" |
| 180 | COLL9 | Bat180 | TROIS RIVIERES | M | 10°38'45.1" | 002°55'41.8" |

M, F: = male or female palm, respectively.

All geographic coordinates are provided as North from the Equator (latitude) and East from the Greenwich meridian (longitude), respectively.
