# Supplementary material for: Transferability, development of simple sequence repeat (SSR) markers and application to the analysis of genetic diversity and population structure of the African fan palm (Borassus aethiopum Mart.) in Benin
Source: BMC Genet. 2020 Dec 3;21:145. doi: 10.1186/s12863-020-00955-y (PMC7713368; doi:10.1186/s12863-020-00955-y)
Supplement: Supplementary file 4 — Additional file 4. Results of the Bayesian cluster analysis with variable values of K. Graphical summary generated from STRUCTURE results by CLUMPAK’s main pipeline with values of K ranging from 1 to 10. [file 12863_2020_955_MOESM4_ESM.pdf]

CLUMPAK main pipeline - Job 1604966026 summary

Major modes for the uploaded data:

K=1

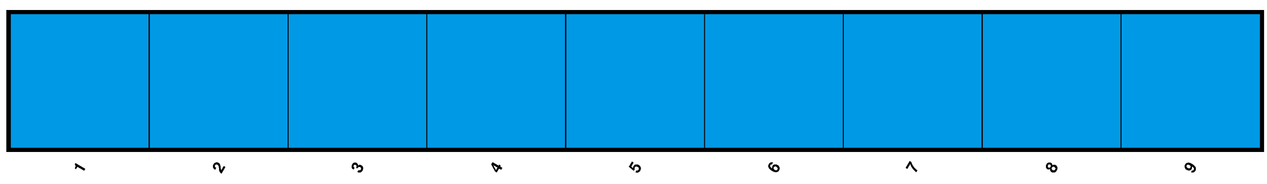

K=2

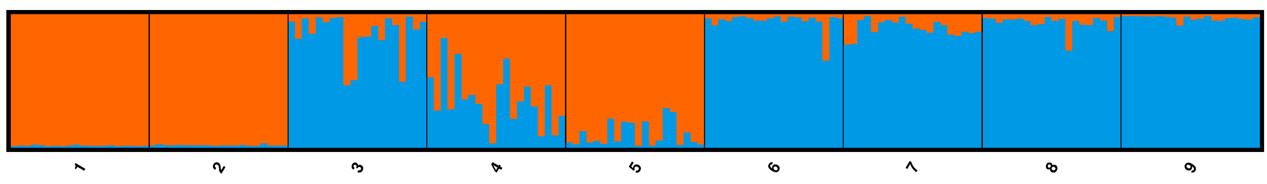

K=3

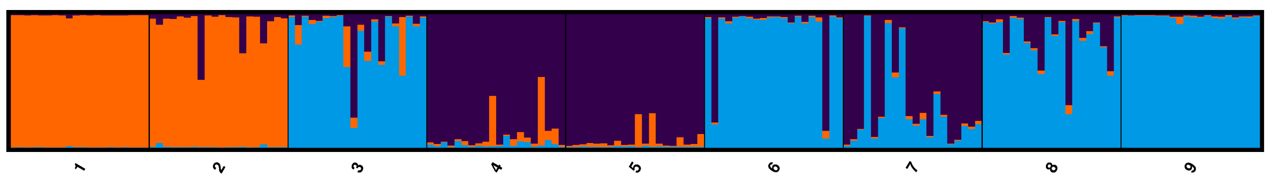

K=4

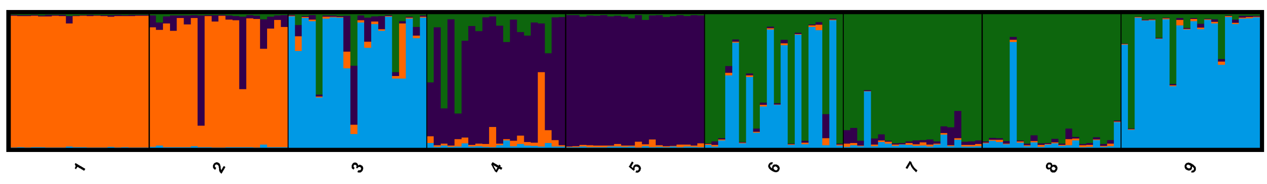

K=5

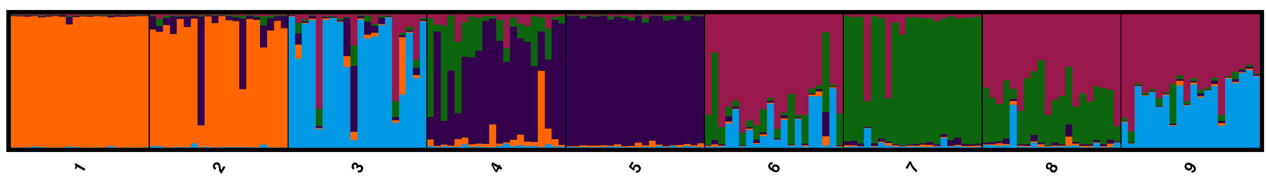

K=6

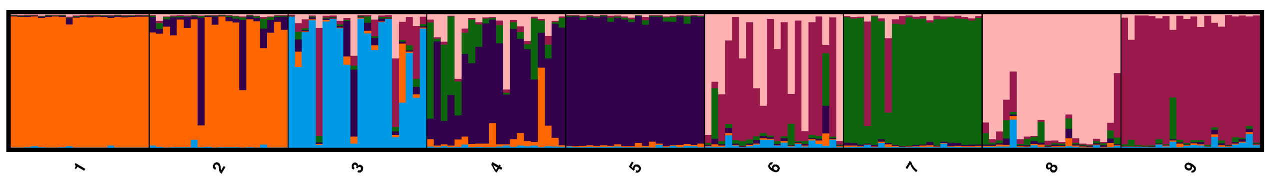

K=7

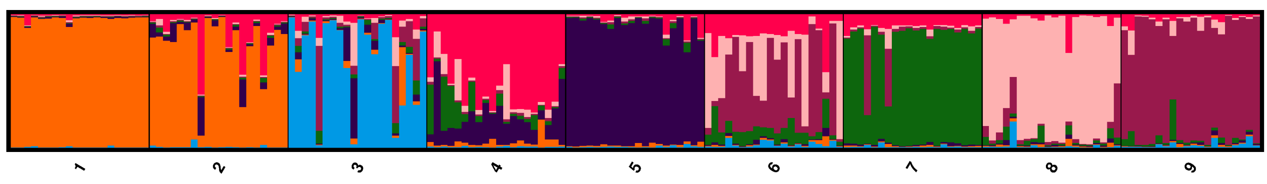

K=8

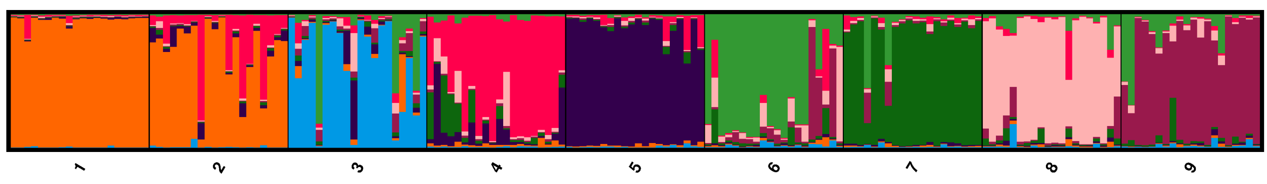

K=9

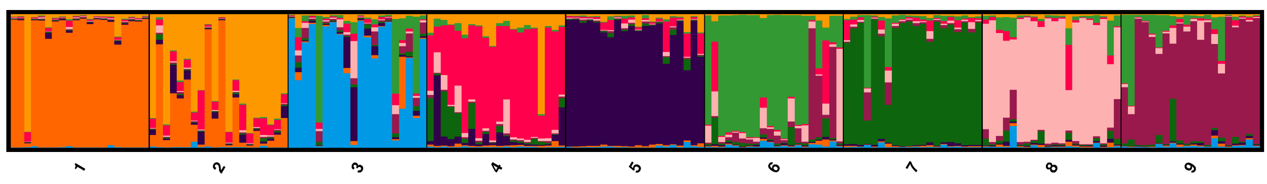

K=10

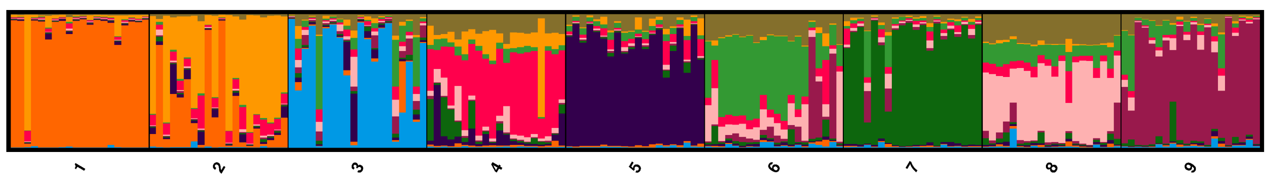

Minor modes for the uploaded data:

K=5 MinorCluster1

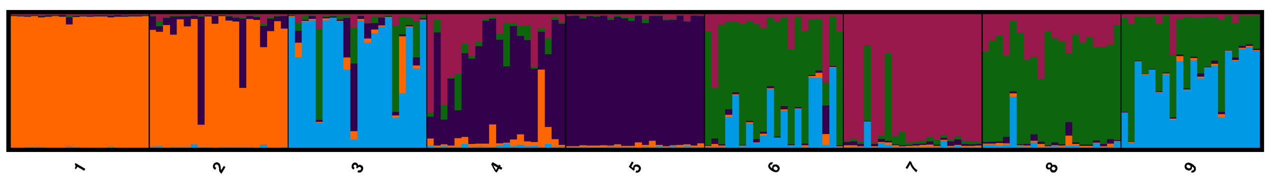

Division of runs by mode:

K=1	6/6
K=2	6/6
K=3	6/6
K=4	6/6
K=5	3/6, 3/6
K=6	6/6
K=7	6/6
K=8	6/6
K=9	6/6
K=10	6/6
